# Atomic Interlayer Mo–N_4_ Sites Enable Rapid Charge Transfer and Efficient CO_2_ Photoreduction

**DOI:** 10.1002/advs.75217

**Published:** 2026-04-13

**Authors:** Lijuan Sun, Haiwei Su, Zhen Chen, William Orbell, Guijie Liang, Weikang Wang, Lele Wang, Juan Yang, Qinqin Liu, Junhua Li

**Affiliations:** ^1^ School of Materials Science and Engineering Jiangsu University Zhenjiang Jiangsu P. R. China; ^2^ State Key Joint Laboratory of Environment Simulation and Pollution Control School of Environment Tsinghua University Beijing P. R. China; ^3^ Hubei Key Laboratory of Low Dimensional Optoelectronic Materials and Devices Hubei University of Arts and Science Xiangyang P.R. China

**Keywords:** covalent organic frameworks, CO_2_ reduction, Mo single atom, interlayer electron transfer, CO_2_ activation

## Abstract

Photocatalytic CO_2_ reduction is a promising route for sustainable carbon conversion, but its efficiency is often limited by poor charge separation and a lack of functional active sites. Here, we address these challenges by constructing an atomic Mo–N_4_ interlayer electron bridge (IEB) within a bipyridine‐based covalent organic framework (COF) via a photoreduction method. Guided by DFT screening, Mo was identified as the optimal metal center, enabling simultaneous CO_2_ activation and ultrafast vertical electron transfer. The resulting Mo@Tp‐Bpy catalyst achieves co‐production rates of 948.0 µmol g^−1^ h^−1^ for CO and 3741.7 µmol g^−1^ h^−1^ for anisaldehyde via coupled CO_2_ reduction and 4‐methoxybenzyl alcohol oxidation, corresponding to 6.2‐fold and 5.0‐fold enhancements over the pristine Tp‐Bpy COF, respectively. Mechanistic studies reveal that the Mo–N_4_ sites facilitate interlayer charge kinetics and lower thermodynamic barriers for both half‐reactions. This work presents a rational atomic‐level strategy for integrating charge management and catalytic function in layered materials toward efficient photoredox catalysis.

## Graphical abstract

This work achieved efficient CO_2_ activation and ultrafast interlayer electron transport in a 2D COF by constructing atomic‐level Mo‐N_4_‐induced interlayer electron bridges (IEB), thereby kinetically and thermodynamically co‐driving the photocatalytic coupling of CO_2_ reduction with 4‐methoxybenzyl alcohol oxidation.

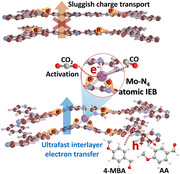

## 1 Introduction

The escalating atmospheric CO_2_ concentration, driven by anthropogenic emissions, alongside the pressing need for sustainable chemical manufacturing, has prompted intensive exploration of solar‐driven photocatalytic systems capable of transforming abundant feedstocks into valuable products [1, 2, 3]. With efficient charge separation, an ideal photocatalyst should be capable of simultaneously driving CO_2_ reduction reaction (CO_2_RR) and its matched oxidation half‐reaction, such as H_2_O or organics oxidation [4, 5, 6, 7]. Among various oxidation targets, 4‐methoxybenzyl alcohol (4‐MBA) is particularly attractive as its selective oxidation produces anisaldehyde (AA), a high‐value aromatic aldehyde widely used in the pharmaceutical, fragrance, and agrochemical industries [8, 9, 10]. Conventional production of AA often involves energy‐intensive processes or stoichiometric oxidants [11], underscoring the need for sustainable alternatives. Integrating 4‐MBA oxidation with CO_2_ reduction in a photocatalytic system not only enhances electron utilization but also simultaneously generates two valuable products, offering a promising route toward green and atom‐efficient chemical synthesis. Nevertheless, the current catalysts are fundamentally constrained by two interrelated limitations: the scarcity of well‐defined active sites that can activate CO_2_ molecules and inefficient charge separation/transport kinetics [12, 13, 14, 15]. These challenges highlight the urgent demand for advanced photocatalysts that combine atomic‐level structural precision with exceptional charge management capabilities.

Materials such as graphitic carbon nitride, metal organic frameworks, and covalent organic frameworks (COFs) possess high specific surface areas, which are beneficial for enhancing CO_2_ absorption [16, 17, 18]. In contrast, transition metal compounds such as copper‐, palladium‐, and molybdenum (Mo)‐based materials exhibit superior capability for CO_2_ activation, owing to their accessible metal ion sites [19, 20, 21]. Single‐atom catalysts (SACs) have recently emerged as an effective strategy to precisely bridge the catalytically active metal sites and functional carrier matrices [22, 23, 24, 25]. Among various supports, 2D imine‐linked COFs—especially those incorporating bipyridine (Bpy) units—offer a distinctive advantage: the positions and orientations of the unsaturated pyridinic N sites are highly predictable and periodic, providing an ideal molecular template for constructing well‐defined surface single metal sites [26, 27, 28]. This characteristic has led to the development of numerous advanced Bpy‐COF‐based SACs with excellent surface chemistry [29].

Despite these advances, a critical yet often overlooked limitation of layered 2D COFs remains their poor interlayer charge transport, which severely restricts the overall photocatalytic efficiency. This limitation originates from the intrinsic constraints of monolayer or restacked systems, where charge separation is fundamentally hampered by rapid in‐plane recombination and the lack of a directional driving force for spatial charge partitioning [30]. To address this challenge, a promising design strategy is to precisely anchor structurally single metal atoms within the interlayer space of COFs. Such a strategy aims to simultaneously achieve two pivotal objectives: (i) establishing an efficient cross‐layer electron transfer pathway to promote charge separation, and (ii) constructing highly active sites at the nodes of this pathway to facilitate CO_2_ activation and conversion. This approach further calls for systematic theoretical screening among various transition metal elements (so far unexplored) to identify the optimal interlayer metal center that can best synergize these dual functions.

In this work, guided by density functional theory (DFT) screening, we report the construction of a Mo atomic interlayer electron bridge (IEB) within a Bpy‐based COF (Mo@Tp‐Bpy) via a facile photoreduction method. Combined experimental and theoretical analyses unambiguously confirm that single Mo atoms are anchored between the layers of Tp‐Bpy COF, forming a well‐defined Mo–N_4_ configuration with the unsaturated pyridinic N sites. Benefiting from this unique Mo‐based IEB, ultrafast vertical interlayer charge transfer and efficient CO_2_ activation are achieved simultaneously. Therefore, Mo@Tp‐Bpy serves as a dual‐functional platform that enables photocatalytic CO_2_ reduction to CO coupled with the selective oxidation of 4‐methoxybenzyl alcohol (4‐MBA) to AA. As a result, co‐production rates of 948.0 and 3741.7 µmol g^−1^ h^−1^ for CO and AA were achieved, respectively, which are 6.2 and 5.0 times higher than those of the pristine Tp‐Bpy COF. Theoretical calculations further reveal that such an atomic IEB can synergistically lower the thermodynamic barriers for both CO_2_ reduction and 4‐MBA oxidation. This work offers new insights into designing efficient layered photocatalysts and achieving cooperative regulation of complex reaction pathways.

## 2 Results and Discussion

### 2.1 Theoretical Screening and Electronic Design of Mo@Tp‐Bpy

**FIGURE 1:**
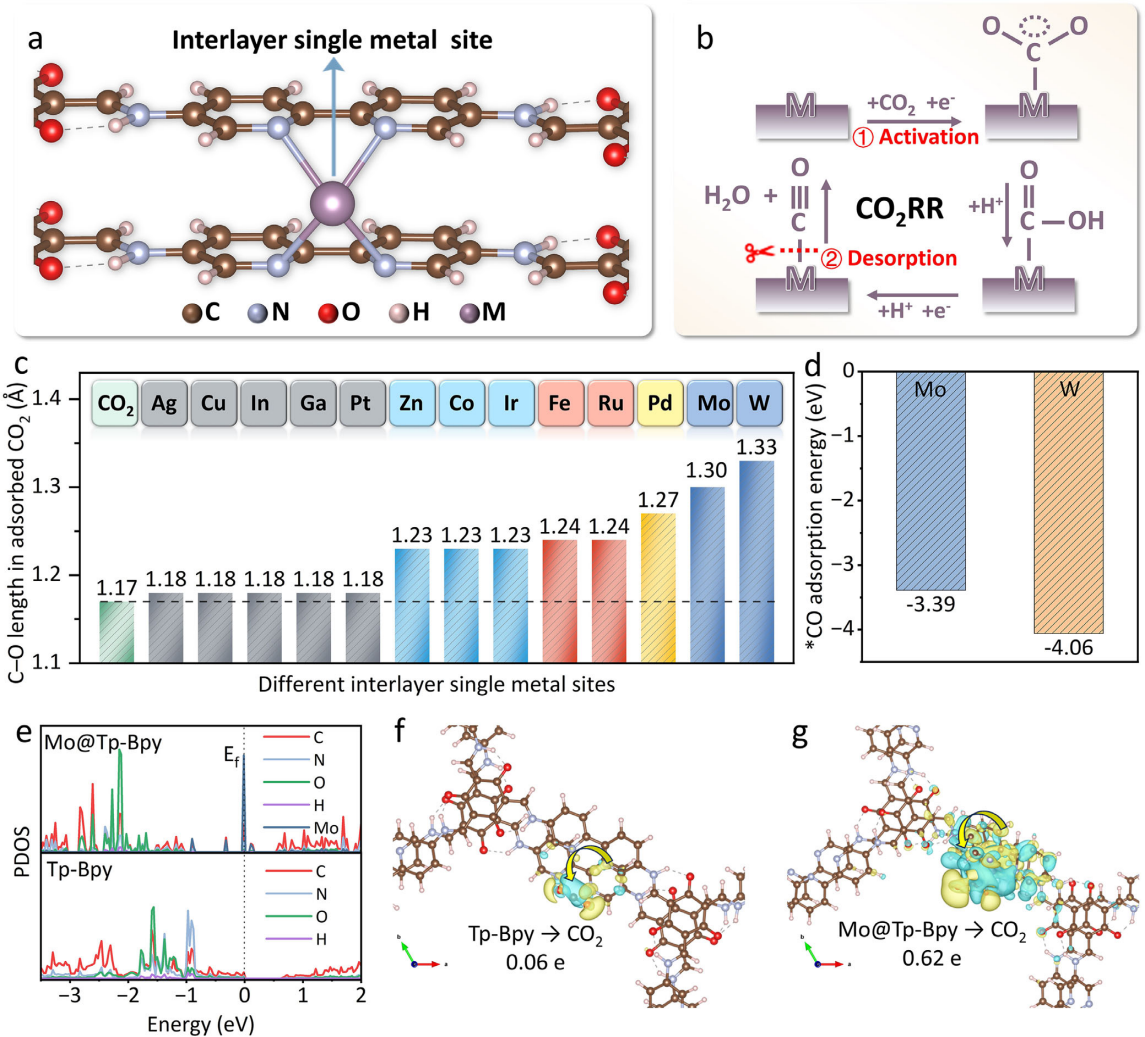
(a) Schematic illustration of atomic‐level bridging between COF layers via anchored metal single atoms. (b) Catalytic cycle for CO_2_ reduction to CO. (c) Elongation of C─O bond length in adsorbed CO_2_ across 13 transition single metal atoms on Tp‐Bpy COF. (d) Comparison of ^*^CO adsorption free energy for Mo and W atoms. (e) PDOS plots of Mo@Tp‐Bpy and pristine Tp‐Bpy COF. Bader charge analysis of CO_2_ adsorbed on the (f) Tp‐Bpy and (g) Mo@Tp‐Bpy.

To rationally design an efficient IEB for photocatalytic CO_2_RR, we first screened potential metal centers anchored between the layers of a bipyridine‐based COF (Figure 1a). The CO_2_RR catalytic cycle involves two critical steps: CO_2_ activation and subsequent ^*^CO desorption (Figure 1b). We systematically optimized the surface structures (Figure S1) and evaluated the CO_2_ activation capability of 13 transition metals by density functional theory (DFT) calculations, where the elongation of the C─O bond length (Figure 1c, while the consistent change of dihedral angle is shown in Figure S2) in adsorbed CO_2_ was adopted as the primary descriptor. Among the candidates, Mo and W exhibited the most pronounced bond elongation, indicating superior CO_2_ activation. However, further analysis of ^*^CO adsorption energy (Figure 1d; Figure S3) revealed that W binds ^*^CO intermediates too strongly (more negative), which would hinder the desorption step and limit the turnover [31]. In contrast, Mo maintains a moderate ^*^CO adsorption strength, thereby balancing activation and product release. Projected density of state (PDOS) analysis shows that introducing Mo single atoms introduces new mid‐gap states and a Mo‐derived energy level crossing the Fermi level, significantly enhancing the electrical conductivity compared to the pristine Tp‐Bpy COF (Figure 1e) [32]. Bader charge analysis further demonstrates that pure Tp‐Bpy COF only transfers 0.06 e^−^ to CO_2_ (Figure 1f), markedly lower than the 0.62 e^−^ donated by the Mo@Tp‐Bpy (Figure 1g). This indicates that the single Mo atom acts as an electron transfer bridge, channeling charge into CO_2_ via Mo–N coordination, thereby laying the electronic foundation for efficient CO_2_ reduction. Therefore, Mo is selected as the optimal IEB center, owing to its favorable CO_2_ activation, suitable ^*^CO adsorption, and enhanced interlayer electron conductivity.

### 2.2 Synthesis and Characterization of Mo@Tp‐Bpy

**FIGURE 2:**
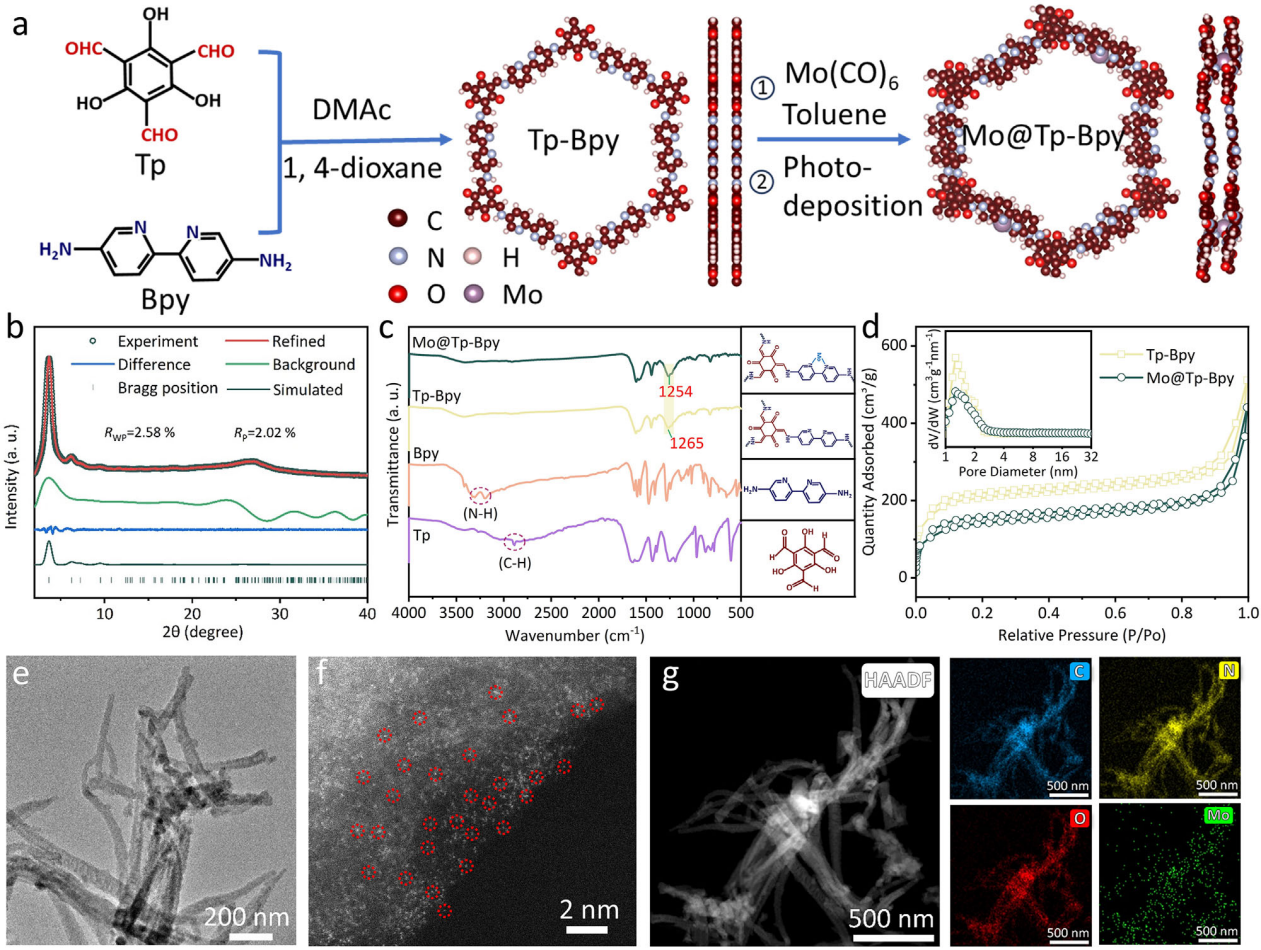
(a) Synthetic strategy and structural models of Mo@Tp‐Bpy single‐atom catalyst. (b) XRD pattern and (c) FT‐IR spectroscopy of Mo@Tp‐Bpy. (d) N_2_ adsorption–desorption isotherms and pore size distribution of Tp‐Bpy and Mo@Tp‐Bpy. (e) TEM image, (f) AC‐HAADF‐STEM image and (g) HAADF‐STEM and elemental mappings of Mo@Tp‐Bpy.

The Mo@Tp‐Bpy catalyst was synthesized through a two‐step procedure, as shown in Figure 2a. First, the Tp‐Bpy COF was constructed via Schiff‐base condensation between 1,3,5‐triformylphloroglucinol (Tp) and 2,2'‐bipyridine‐5,5'‐diamine (Bpy). The periodic topology and ordered channels of the resulting COF, together with the well‐defined bipyridine coordination sites, provide an ideal platform for anchoring high oxidation state metal atoms. Subsequently, Mo atoms were anchored via a solvothermal‐assisted photoreduction of Mo(CO)_6_ in toluene, yielding the Mo@Tp‐Bpy catalyst. The actual Mo loading amount was determined by inductively coupled plasma mass spectrometry (ICP‐MS) (Figure S4). The crystallinity of the as‐synthesized material was verified by X‐ray diffraction (XRD, Figure 2b). The pattern exhibits characteristic peaks at 3.7° and 6.1°, corresponding to the (100) and (200) diffraction planes of Tp‐Bpy COF, respectively. A broad peak observed at a higher 2θ value of ∼26.6° is attributed to the (001) plane reflection, arising from interlayer π–π stacking [33]. Excellent agreement between the experimental pattern and the refined structural model, with low R_wp_ and R_p_ values of 2.58% and 2.02%, respectively, confirms the high phase purity and structural retention of the Tp‐Bpy COF architecture after Mo incorporation, consistent with the simulated and pristine Tp‐Bpy patterns (Figure S5). The refined unit cell parameters (a = b = 30.53 Å, c = 3.50 Å, α = β = 90°, γ = 120°) further corroborate the well‐defined crystalline structure. Further structural evidence was provided by Fourier‐transform infrared (FT‐IR) spectroscopy (Figure 2c). The disappearance of the aldehyde C═O stretch (1637 cm^−1^) from Tp and N–H vibrations from Bpy, along with the emergence of characteristic C═N (1606 cm^−1^) and C─N (1265 cm^−1^) stretching modes in Tp‐Bpy, confirms a successful Schiff‐base condensation process [34]. In Mo@Tp‐Bpy, a noticeable shift of the C–N vibration to 1254 cm^−1^ suggests coordination between the introduced Mo atoms and the bipyridine N sites. This red shift indicates a weakening of the C─N bond, consistent with electron density donation from the N lone pair to the Mo center. Solid‐state ^13^C NMR spectra (Figure S6) further revealed that Mo does not coordinate with C atoms due to the absence of significant chemical shift changes in Mo@Tp‐Bpy.

N_2_ adsorption–desorption measurements were used to evaluate the porosity and specific surface area of the catalysts (Figure 2d). Both Tp‐Bpy and Mo@Tp‐Bpy exhibit a sharp uptake at low relative pressures (P/P_0_< 0.05), characteristic of microporous materials [35]. The Brunauer–Emmett–Teller (BET) surface area decreases from 780.3 m^2^ g^−1^ for Tp‐Bpy to 536.5 m^2^ g^−1^ for Mo@Tp‐Bpy, while the Non‐local Density Functional Theory (NLDFT) derived pore size distribution (inset) remains nearly identical. This preserved pore architecture alongside a reduced surface area and total pore volume (Table S1), which provides strong evidence for the successful and uniform incorporation of Mo single atoms within the Tp‐Bpy COF channels. Scanning electron microscopy (SEM) images of Tp‐Bpy show an interwoven nanofiber morphology (Figure S7a), and it is well‐preserved in Mo@Tp‐Bpy, indicating that the introduction of Mo single atoms does not compromise the structural integrity of the Tp‐Bpy COF (Figure S7b). This is also corroborated by transmission electron microscopy (TEM, Figure 2e and Figure S8), which reveals the maintained nanofiber structure of Mo@Tp‐Bpy. High‐resolution TEM image shows no observable nanoparticles or clusters, providing additional evidence for the atomic dispersion of Mo species (Figure S9). Direct observation of atomic dispersion is provided by chromatic aberration‐corrected high‐angle annular dark‐field scanning transmission electron microscopy (AC‐HAADF‐STEM, Figure 2f), where bright spots (highlighted in red) correspond to isolated Mo atoms. Furthermore, the elemental mappings (Figure 2g) confirm the homogeneous spatial distribution of C, N, O, and Mo throughout the COF, verifying the successful integration of Mo species within the COF.

### 2.3 Coordination Environment and Structural Parameter of Mo@Tp‐Bpy

**FIGURE 3:**
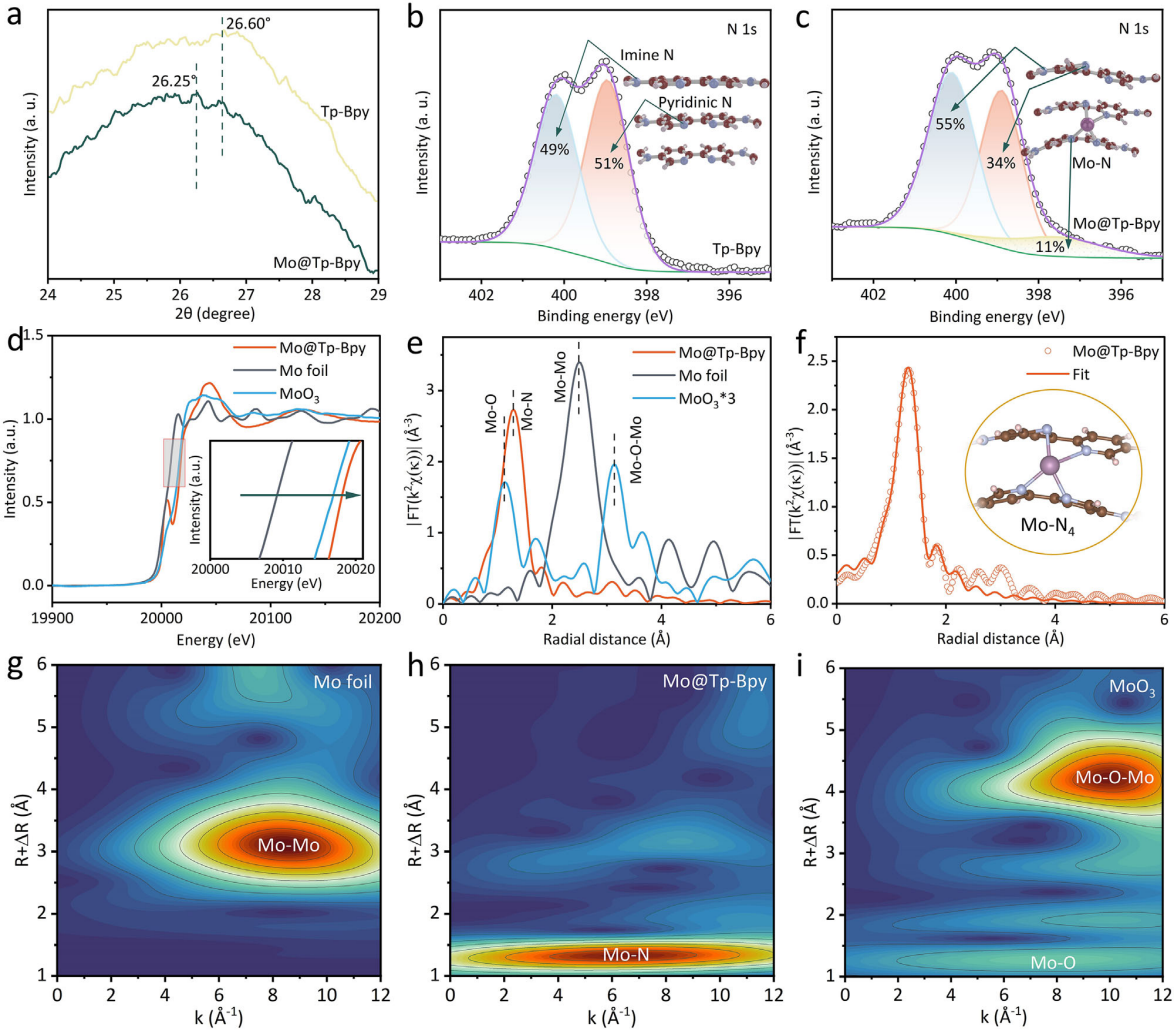
(a) Slow‐scan XRD patterns of Tp‐Bpy and Mo@Tp‐Bpy. N 1s XPS analysis of (b) Tp‐Bpy and (c) Mo@Tp‐Bpy. (d) XANES spectra and (e) FT‐EXAFS spectra of Mo@Tp‐Bpy, Mo foil, and MoO_3_. (f) EXAFS fitting and structural model of Mo@Tp‐Bpy. WT‐EXAFS spectra for (g) Mo foil, (h) Mo@Tp‐Bpy, and (i) MoO_3_.

The interlayer spacing of Tp‐Bpy and Mo@Tp‐Bpy was characterized by slow‐scan XRD patterns (24°–29°) (Figure 3a). The (001) diffraction peak of Tp‐Bpy in Mo@Tp‐Bpy significantly shifted from 26.60° to 26.25° compared to the pristine Tp‐Bpy. The decrease in the diffraction angle indicates that the introduction of Mo led to an expansion of the interlayer spacing [36]. X‐ray photoelectron spectroscopy (XPS) was employed to probe the valence state and electronic structure of the catalysts (Figure S10). The N 1s signal in Tp‐Bpy COF sample (Figure 3b) can be deconvoluted into two components at 398.9 and 400.2 eV, corresponding to pyridinic N and imine N, respectively [37]. In contrast (Figure 3c), a new peak emerges at 397.4 eV over Mo@Tp‐Bpy, assigned to Mo–N bond, accompanied by a decrease in the intensity of the pyridinic N peak at 398.9 eV from 51% to 34%. Such a decreasing shift provides direct evidence for electron transfer from pyridinic N to Mo during coordination. To explore the spatial distribution of Mo species, depth profiling was conducted via gradual Ar^+^ sputtering to remove the surface layers. The Mo content was observed to increase from 14.87 to 17.77 at% with prolonged sputtering time (Figure S11, Table S2), indicating a concentration gradient from the catalyst surface to the bulk [38]. This result confirms that the Mo atoms are incorporated within the interlayers of Tp‐Bpy, corroborating the XRD results, which suggested interlayer expansion.

X‐ray absorption spectroscopy (XAS) measurements were conducted to investigate the local coordination environment of Mo atoms. The Mo K‐edge X‐ray absorption near‐edge structure (XANES) spectrum (Figure 3d) shows that the absorption near‐edge position of Mo@Tp‐Bpy aligns closely with that of MoO_3_, confirming the high oxidation state of Mo species [39]. In the Fourier transform extended X‐ray absorption fine structure (FT‐EXAFS, Figure 3e), Mo@Tp‐Bpy only exhibits a single prominent peak located at ∼1.3 Å, attributed to Mo–N scattering [40, 41], with no detectable Mo–Mo or Mo–O contributions, indicating the absence of Mo clusters or oxides. EXAFS fitting (Figure 3f, Figure S12, and Table S3) reveals a coordination number of ∼4 N atoms around Mo, consistent with the simulated structural model (inset in Figure 3f). Further validation comes from the wavelet transform (WT) analysis (Figure 3g–i). While Mo foil shows a strong Mo–Mo contribution, and MoO_3_ exhibits intensity maxima corresponding to Mo–O and Mo–O–Mo paths. Notably, the WT plot of Mo@Tp‐Bpy displays only one intensity maximum attributable to Mo–N coordination, reinforcing the conclusion that Mo is atomically dispersed and coordinated exclusively by N atoms. These results suggest that the Mo–N_4_ center is likely coordinated by pyridinic N atoms from both adjacent COF layers, effectively creating an IEB.

### 2.4 Ultrafast Charge Transfer Kinetics Analysis

**FIGURE 4:**
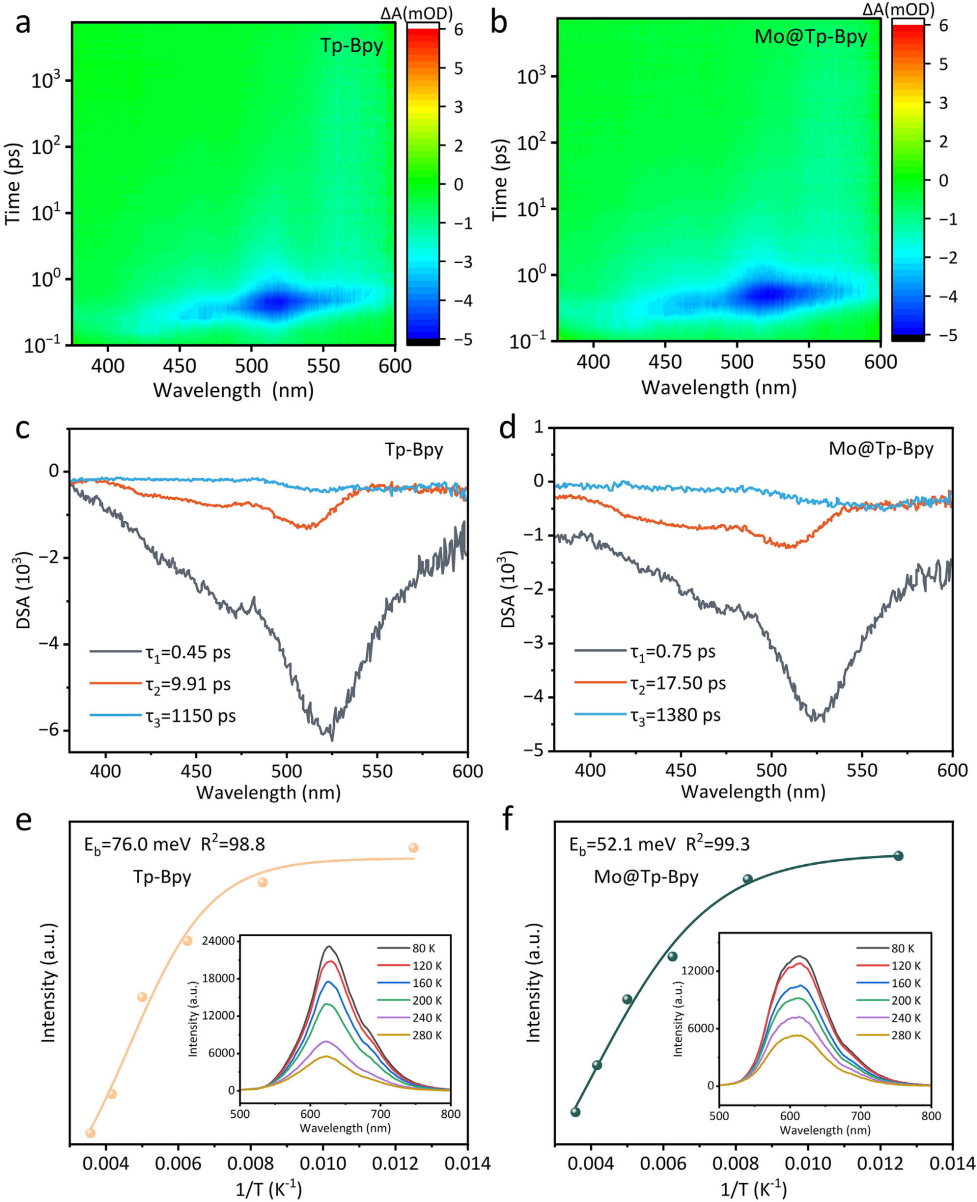
fs‐TAS contour plot of (a) Tp‐Bpy and (b) Mo@Tp‐Bpy. Corresponding decay‐associated spectra of (c) Tp‐Bpy and (d) Mo@Tp‐Bpy. Temperature‐dependent PL and Arrhenius analysis of (e) Tp‐Bpy and (f) Mo@Tp‐Bpy (inset: PL spectra).

The enhanced charge transport efficiency mediated by IEB in Mo@Tp‐Bpy was investigated through photoelectrochemical measurements, including photocurrent response, electrochemical impedance spectroscopy (EIS) plots, photoluminescence (PL) spectra, time‐resolved PL (TR‐PL) spectra, and transient photovoltage (TPV) (Figures S13–S16 and Table S4). [42] The charge transfer dynamics in Mo@Tp‐Bpy were directly interrogated using femtosecond transient absorption spectroscopy (fs‐TAS). As shown in Figure 4a,b, both Tp‐Bpy and Mo@Tp‐Bpy exhibit similar spectral shapes in the 400–600 nm region, consistent with ground state bleaching (GSB) dominated processes. [43] The fs‐TAS intensity in each sample reached a maximum at 0.5 ps and subsequently decayed over time. Global fitting analysis results indicate that the three excited state species (DAS) exhibit spectral information with characteristic peaks centered at approximately 470, 520, and 575, respectively. Among these, the negative signal intensity at 520 nm is the strongest. According to the apparent quantum efficiency (AQE) and UV–vis measurement results (Figure S17), the signals at 470 and 520 nm can be attributed to the intrinsic absorption of Mo@Tp‐Bpy. In contrast, the weak negative feature observed at 575 nm may originate from the combined contribution of residual GSB and stimulated emission (fluorescence effects). Here, we assign the first excited state to singlet excitons. Meanwhile, the charge‐transfer state of the second excited state, modulated by the electronic environment of the Mo element, accelerates interlayer charge transfer, resulting in a longer‐lived charge‐transfer state. Finally, the signal at 575 nm is attributed to the lowest excited state dominated by radiative processes.

For pristine Tp‐Bpy COF, the dynamics were also well described with time constants τ_1_ = 0.45 ps, τ_2_ = 9.91 ps and τ_3_ = 1150 ps (Figure 4c). The shortest component (τ_1_) is attributed to the lattice diffusion process of photogenerated charges, the intermediate component (τ_2_) to electron trapping at defect sites or shallow trap states, and the longest component (τ_3_) to the direct recombination of HOMO holes. [36, 44] For Mo@Tp‐Bpy, the decay kinetics were fitted by three distinct lifetime components: τ_1_ = 0.75 ps, τ_2_ = 17.50 ps, and τ_3_ = 1380 ps (Figure 4d). Compared to Tp‐Bpy COF, the emergence of a more pronounced intermediate component (τ_2_ = 17.50 ps) in Mo@Tp‐Bpy is associated with efficient electron trapping at the Mo atomic sites. In Mo@Tp‐Bpy, the Mo atomic IEB can instantaneously capture surrounding photogenerated electrons, giving rise to this additional decay pathway and the observed increase in the carrier lifetime. Overall, the construction of interlayer Mo–N_4_ sites creates an additional electron trapping pathway, effectively suppressing electron‐hole recombination and thus favoring the photocatalytic performance (Figure S18). In situ irradiation X‐ray photoelectron spectroscopy (ISI‐XPS) further corroborated the photoinduced charge transfer, revealing a negative shift of Mo 3d peaks upon illumination, which confirms electron accumulation at Mo sites to drive the subsequent CO_2_ reduction (Figure S19).

To further understand the origin of the prolonged carrier lifetime in Mo@Tp‐Bpy, temperature‐dependent PL studies were conducted to extract the exciton binding energy (E_a_). As shown in Figure 4e,f, the temperature‐dependent PL intensity was analyzed using the equation:

(1) $$I\left(T\right)=I_{0}\;/\left(1+Ae^{-E_{b}/k_{B}T}\right)$$

where I_0_ is the intensity at 0 K, k_B_ is the Boltzmann constant, and k_B_T ∼23 meV at room temperature [45]. Tp‐Bpy exhibits an E_a_ of 76.0 meV (R^2^ = 98.8%), which decreases markedly to 52.1 meV (R^2^ = 99.3%) for Mo@Tp‐Bpy. The marked reduction in E_a_ confirms that the incorporation of atomic IEB significantly facilitates exciton dissociation into free electrons and holes. Consequently, Mo@Tp‐Bpy exhibits accelerated charge transfer and enhanced separation efficiency, concurrently promoting hole accumulation on the framework, which is highly favorable for driving coupled redox cooperative reactions.

### 2.5 Photocatalytic CO_2_ Reduction Coupled with 4‐MBA Oxidation Performance

**FIGURE 5:**
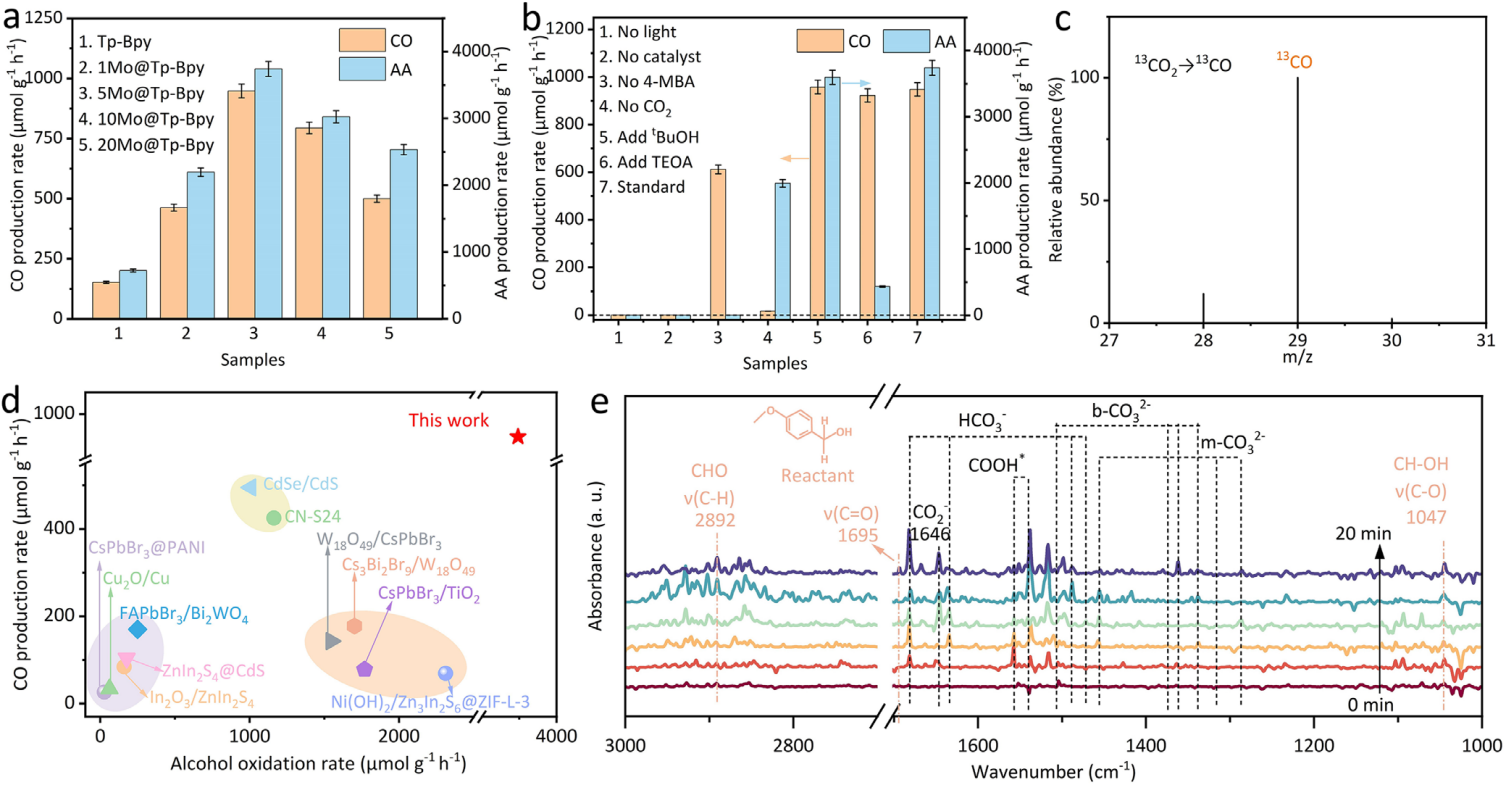
(a) Photocatalytic CO and AA production rates of different samples. (b) Control experiments under different conditions on Mo@Tp‐Bpy. (c) MS analysis of gas products during the reaction catalyzed by Mo@Tp‐Bpy. (d) The comparison of this work with the recently reported advanced photocatalysts toward coupled CO_2_ reduction and alcohols oxidation reaction. (e) In situ DRIFTS spectra of the photoreaction process on Mo@Tp‐Bpy.

The photocatalytic performance of the as‐synthesized catalysts was systematically evaluated in a coupled reaction system for CO_2_ reduction to CO and the simultaneous oxidative coupling of 4‐MBA to AA. As illustrated in Figure S20, the CO production rate over the catalysts exhibited a strong dependence on the Mo loading amount. The oxidation product concentrations were determined by GC analysis using an external method (Figure S21 and Table S5). The optimal catalyst was Mo@Tp‐Bpy with 5% Mo content, achieved a remarkable CO production rate of 948.0 µmol g^−1^ h^−1^, which was approximately 6.2 times higher than that of the pristine Tp‐Bpy (152.5 µmol g^−1^ h^−1^). The production rate of AA over Mo@Tp‐Bpy reached an impressive 3741.7 µmol g^−1^ h^−1^, a more than fivefold enhancement compared to Tp‐Bpy (722.3 µmol g^−1^ h^−1^) (Figure 5a). To confirm the origin of the photocatalytic products, a series of control experiments was meticulously conducted (Figure 5b). Negligible product formation is observed in the absence of light, catalyst, CO_2_, or 4‐MBA. The AQE of Mo@Tp‐Bpy reaches 3.87% at 420 nm (Figure S17), affirming efficient visible‐light utilization. Isotope labeling experiments using ^13^CO_2_ and subsequent GC–MS detection of ^13^CO (m/z = 29, Figure 5c) verify that the produced CO originates predominantly from CO_2_ feedstock. Cycling tests (Figure S22) demonstrated excellent stability of the Mo@Tp‐Bpy catalyst, retaining over 92% of its initial activity for both CO and AA production over five consecutive runs. Post‐reaction characterizations confirm the preserved structural integrity of Mo@Tp‐Bpy. (Figures S23–S25), which underscores the catalyst's significant potential for practical application as a durable and reusable catalyst. Compared to currently advanced photocatalysts with similar coupling reactions (Figure 5d, Table S6), Mo@Tp‐Bpy exhibited the comprehensive advantages of bifunctional activity in the coupled CO_2_ reduction and alcohol oxidation reaction, highlighting its prowess in coupled photocatalysis and the design merit of structure‐performance synergy.

In addition, removing 4‐MBA drastically suppresses CO generation, while replacing CO_2_ with N_2_ markedly lowers AA yield, indicating that the oxidation and reduction processes are synergistically coupled. The addition of tert‐butanol (^t^BuOH), a hydroxyl radical scavenger, shows little effect on the formation of AA or CO, ruling out the ·OH‐mediated oxidation pathway [46]. In contrast, triethanolamine (TEOA), a hole scavenger, significantly inhibits AA production, confirming that photogenerated holes are essential for oxidizing 4‐MBA. Furthermore, in situ diffuse reflectance infrared Fourier transform spectroscopy (DRIFTS) was employed to directly monitor the reactive intermediates and unravel the temporal production of both half‐reactions (Figure 5e). Characteristic peaks for CO_2_^−^ (at 1646 cm^−1^) and bicarbonate species, HCO_3_^−^ (at 1683, 1635, 1488, and 1473 cm^−1^), were observed to emerge. Peaks at 1454, 1317, and 1287 cm^−1^ were identified as monodentate carbonate (m‐CO_3_^2−^), while those at 1508, 1338, 1373, and 1360 cm^−1^ correspond to bidentate carbonate (b‐CO_3_^2−^). Importantly, two distinct signals at 1557 and 1540 cm^−1^ are attributed to COOH^*^, confirming its role as a key intermediate in CO_2_‐to‐CO conversion [47, 48, 49]. Simultaneously, the gradual intensification of peaks at 1695 and 2892 cm^−1^, assigned to C═O and C─H stretching of the aldehyde group, respectively, reflects the continuous formation of AA from 4‐MBA oxidation. An emerging absorption at 1047 cm^−1^, associated with C─O stretching in ^*^RCH(OH), becomes more pronounced over time, indicating initial C─H bond activation. Furthermore, in situ DMPO‐trapped EPR spectroscopy was conducted to directly confirm the generation of radical species in the presence of 4‐MBA. As illustrated in Figure S26, a set of six enhanced signals assignable to carbon‐centered radicals was observed, which have been widely recognized as key intermediates in the photocatalytic alcohol oxidation reaction [50]. This finding provides strong evidence that the C─H bond in 4‐MBA is primarily cleaved via oxidative activation by holes, which is consistent with the scavenger experiments and DRIFTS results.

### 2.6 Mechanistic Insights and DFT Calculations

Theoretical simulations unveiled the underlying mechanism responsible for the superior photocatalytic performance. As illustrated in Figure S27, the HOMO and LUMO electron densities in pristine Tp‐Bpy are uniformly delocalized across the framework, suggesting that photogenerated electron–hole pairs remain largely confined, which underlies its large exciton binding energy and high recombination rate. In contrast, Mo@Tp‐Bpy exhibits a marked redistribution of orbital density; the HOMO localizes around Mo and its coordinated N atoms, while the LUMO also concentrates toward the Mo center. This redistribution arises from hybridization between Mo *d*‐orbitals and N *p*‐orbitals, establishing an electronic interaction that facilitates ultrafast charge separation and interlayer electron migration upon photoexcitation. Combined with the experimental results, this orbital reconstruction provides a molecular‐level basis for the cooperative “CO_2_ activation‐electron transfer” mechanism.

**FIGURE 6:**
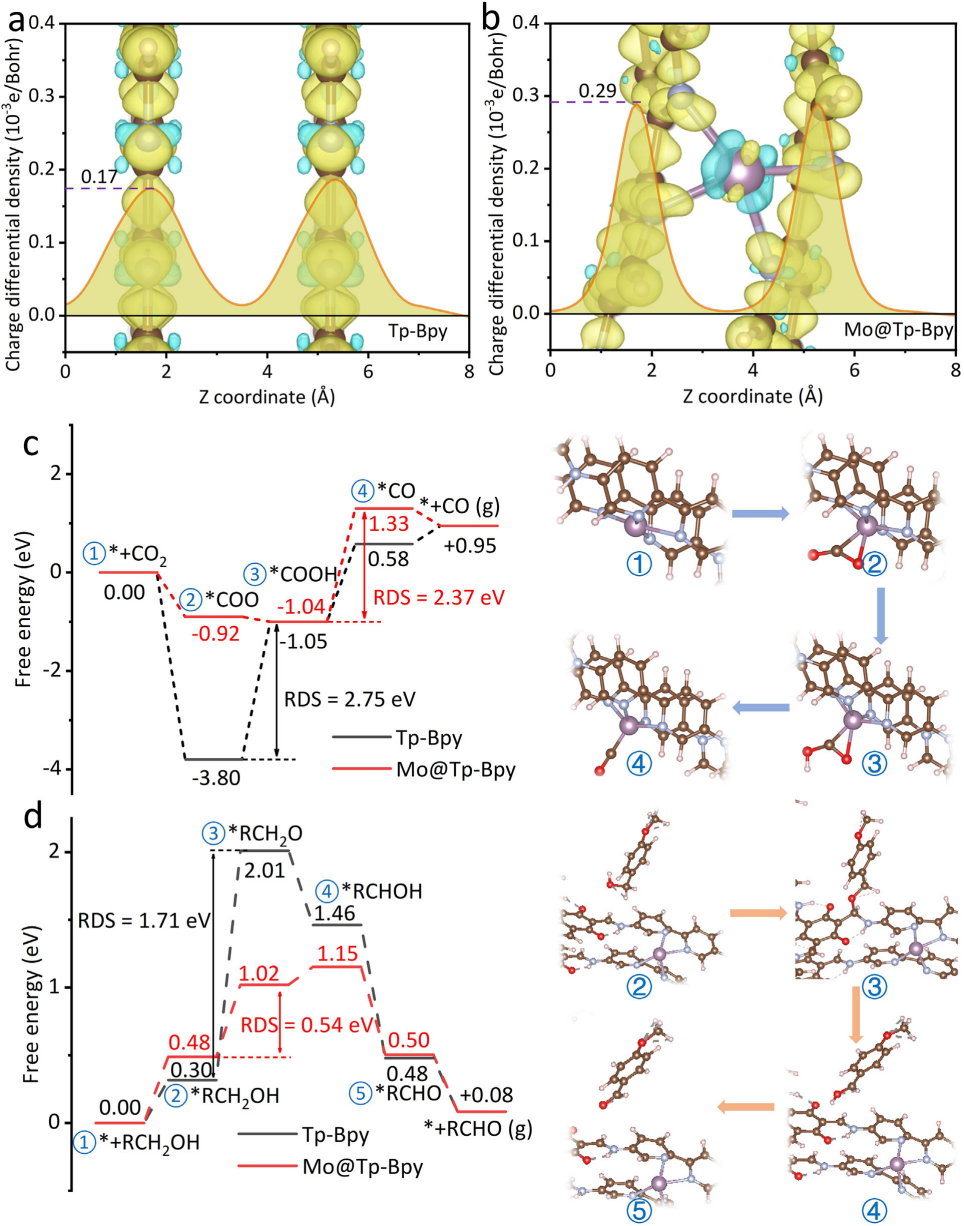
Calculated 1D and 3D (inset) charge density difference distribution (unit in e/Bohr) of (a) Tp‐Bpy and (b) Mo@TP‐Bpy. Free‐energy profiles and corresponding structural configuration over Tp‐Bpy and Mo@Tp‐Bpy for (c) CO_2_‐to‐CO photoreduction and (d) 4‐MBA‐to‐AA Photooxidation.

Unveiling the electronic structure modulation, we performed charge density difference (CDD) and electron localization function (ELF) analyses for both Mo@Tp‐Bpy and Tp‐Bpy. As illustrated in Figure S28, the 1D and 3D (insets) CDD profiles, along with 2D CDD maps and ELF distributions, reveal pronounced electronic modulation induced by Mo atomic IEB. In pristine Tp‐Bpy, the CDD curve along the z‐direction exhibits a modest peak of 0.17 × 10^−3^ e/Bohr^3^ (Figure 6a), consistent with the intrinsic framework charge distribution. In contrast, Mo@Tp‐Bpy shows a significantly enhanced CDD peak of 0.29 × 10^−3^ e/Bohr^3^ (Figure 6b), indicative of pronounced surface layer charge accumulation driven by Mo–N coordination. Furthermore, the influence of interfacial chemical bonding was elucidated by calculating the ELF (Figure S29). In pristine Tp‐Bpy, the C/N sites exhibit a homogeneous distribution of localized charge. With the introduction of Mo, a certain degree of electron delocalization emerges around the Mo centers. The electron density of atoms adjacent to Mo exhibits significant polarization, indicating the formation of new covalent interactions between Mo and N atoms [51]. Moreover, charge accumulation is observed around the neighboring N atoms, further demonstrating that the Mo‐mediated charge redistribution facilitates interlayer charge transport.

In addition, the incorporation of Mo has narrowed the bandgap while ensuring a matched potential alignment for CO_2_ reduction and 4‐MBA oxidation (Figure S30), thereby providing sufficient thermodynamic driving force. The calculated free energy diagrams were simulated to quantitatively compare the energy barriers for both half‐reactions [46, 52]. We first systematically investigated the potential adsorption sites for CO_2_ on Mo@Tp‐Bpy, including the Mo single atoms and the pyridinic N site. After structural optimization, CO_2_ desorbs from N sites. In contrast, at Mo SAs sites, CO_2_ adopts a bidentate coordination involving both C and O atoms, accompanied by significant geometric bending and electronic rearrangement (Figure S31). For CO_2_ reduction (Figure 6c), the rate‐determining step (RDS) on Mo@Tp‐Bpy is the transformation of ^*^COOH to ^*^CO, with a barrier of 2.37 eV, substantially lower than the 2.75 eV required for the RDS (^*^COO→^*^COOH) on Tp‐Bpy. That is, Mo‐N_4_ sites reconfigure the reaction pathway, thereby optimizing the overall CO_2_ conversion energetics. Moreover, ^*^CO desorption becomes exothermic on Mo@Tp‐Bpy, promoting active‐site regeneration and consistent with the high and stable CO production observed. For 4‐MBA oxidation, we first systematically investigated the initial dehydrogenation step to identify the preferred reaction pathway. Interestingly, our DFT calculations reveal that O─H bond cleavage (^*^RCH_2_OH → ^*^RCH_2_O) exhibits a lower energy barrier (ΔG = 0.54 eV) compared to C─H bond cleavage (^*^RCH_2_OH → ^*^RCHOH, ΔG = 0.67 eV) on Mo@Tp‐Bpy (Figure 6d; Figure S32), indicating that O–H activation is the thermodynamically favored initial step. This differs from gas‐phase bond dissociation energies [46], likely because our model accounts for surface interactions with the COF framework. Although O–H cleavage would theoretically generate an oxygen‐centered radical (^*^RCH_2_O), the structural models show that such unsaturated O atoms readily coordinate with the Tp‐Bpy framework, forming stable adsorption configurations that render this oxygen radical undetectable. Instead, a spin‐center transfer‐mediated pathway might be followed [53], where the initially formed ^*^RCH_2_O undergoes 1,2‐hydrogen migration to generate the carbon‐centered radical ^*^RCHOH, which then dehydrogenates to AA. This pathway is fully consistent with the in‐situ EPR results (Figure S26), which show characteristic signals of carbon‐centered radicals. The rate‐determining step for overall oxidation exhibits a lower ΔG on Mo@Tp‐Bpy (0.54 eV) than on pristine Tp‐Bpy COF (1.71 eV), confirming that Mo single atoms lower the dehydrogenation barrier and enhance oxidation efficiency. This enhancement is attributed to the Mo sites acting as efficient electron acceptors for CO_2_ reduction, which continuously withdraws photogenerated electrons from the COF framework, thereby promoting hole accumulation on the organic backbone and facilitating the oxidative dehydrogenation process. These results collectively elucidate the synergistic mechanism in Mo@Tp‐Bpy, the Mo single atoms facilitate CO_2_ activation and reduction, while their primary role in the oxidation half‐reaction is to efficiently extract photogenerated electrons from the framework. This ultrafast electron extraction significantly enhances hole concentration and longevity on the COF matrix, which in turn drives the oxidative dehydrogenation of 4‐MBA directly on the organic framework.

## 3 Conclusions

In summary, we have developed a Mo single‐atom catalyst anchored in a bipyridine‐based COF (Mo@Tp‐Bpy) guided by theoretical screening, which, upon anchoring, forms a well‐defined Mo–N_4_ configuration bridging adjacent COF layers. This atomic IEB serves a dual function: it acts as an efficient site for CO_2_ activation while enabling ultrafast vertical electron transfer. The interlayer Mo–N_4_ sites create directional electron transfer pathways between COF layers, as directly evidenced by a series of photoelectrochemical studies. This unique architecture enables Mo centers to exclusively facilitate CO_2_ activation and reduction, while simultaneously promoting hole accumulation on the framework for efficient oxidative dehydrogenation of 4‐MBA. Combined with in situ spectroscopy and DFT results that verify the reduced energy barriers for both half‐reactions, the catalyst achieves significantly enhanced production rates of 948.0 µmol g^−1^ h^−1^ for CO and 3741.7 µmol g^−1^ h^−1^ for AA, along with excellent stability. This work demonstrates the advantage of interlayer active sites in 2D photocatalysts, providing a new design strategy for efficient solar‐driven coupled redox reactions.

## Funding

This work was supported by the National Science Foundation of China (225B2605, 22102064, and 22472069)

## Conflicts of Interest

The authors declare no conflict of interest.

## Supporting information




**Supporting File**: advs75217‐sup‐0001‐SuppMat.docx

## Data Availability

The data that support the findings of this study are available from the corresponding author upon reasonable request.
