# Supplementary material for: Atomic Interlayer Mo–N4 Sites Enable Rapid Charge Transfer and Efficient CO2 Photoreduction
Source: Adv Sci (Weinh). 2026 Apr 13:e75217. Online ahead of print. doi: 10.1002/advs.75217 (PMC13334637; doi:10.1002/advs.75217)
Supplement: Supplementary file 1 — Supporting File: advs75217‐sup‐0001‐SuppMat.docx [file ADVS-9999-e75217-s001.docx]

**Supporting Information**

**Atomic Interlayer Mo–N_4_ Sites Enable Rapid Charge Transfer and Efficient CO_2_ Photoreduction**

Lijuan Sun,^[a,†]^ Haiwei Su,^[b,†]^ Zhen Chen,^[b,*]^ William Orbell,^[b]^ Guijie Liang,^[c]^ Weikang Wang,^[a,*]^ Lele Wang,^[a]^ Juan Yang,^[a]^ Qinqin Liu,^[a,*]^ Junhua Li^[b,*]^

[a] L. Sun, Dr. W. Wang*, Dr. L. Wang, Prof. J. Yang, Prof. Q. Liu*
School of Materials Science and Engineering, Jiangsu University
Zhenjiang, Jiangsu, 212013, P. R. China
E-mail: qqliu@ujs.edu.cn, wangwk@ujs.edu.cn

[b] H. Su, Dr. Z. Chen*, W. Orbell, Prof. J. Li*
State Key Joint Laboratory of Environment Simulation and Pollution Control, School of Environment, Tsinghua University
Beijing, 100084, P. R. China

E-mail: cz2019@mail.tsinghua.edu.cn, lijunhua@tsinghua.edu.cn

[c] Prof. G. Liang
Hubei Key Laboratory of Low Dimensional Optoelectronic Materials and Devices, Hubei University of Arts and Science
Xiangyang 441053, P.R. China

[^†^] These authors contributed equally to this work.

**Table of content**

[Experimental Section 4](#_Toc224506645)

[Chemicals 4](#_Toc224506646)

[Synthesis of Tp-Bpy COF 4](#_Toc224506647)

[Synthesis of Mo@Tp-Bpy COF 4](#_Toc224506648)

[Characterization 5](#_Toc224506649)

[Photoelectrochemical Measurements 6](#_Toc224506650)

[Electron Paramagnetic Resonance (EPR) 7](#_Toc224506651)

[Femtosecond transient absorption (fs-TA) test 7](#_Toc224506652)

[In situ diffuse reflectance infrared Fourier transform spectroscopy (In situ DRIFTS) test 8](#_Toc224506653)

[Photocatalytic CO_2_ reduction and 4-methoxybenzyl alcohol (4-MBA) oxidation measurement 8](#_Toc224506654)

[DFT calculation details 9](#_Toc224506655)

[The equations for calculating bandgap, HOMO and LUMO positions 9](#_Toc224506656)

[Supplementary Figures 11](#_Toc224506657)

[Figure S1 11](#_Toc224506658)

[Figure S2 12](#_Toc224506659)

[Figure S3 13](#_Toc224506660)

[Figure S4 14](#_Toc224506661)

[Figure S5 15](#_Toc224506662)

[Figure S6 16](#_Toc224506663)

[Figure S7 17](#_Toc224506664)

[Figure S8 18](#_Toc224506665)

[Figure S9 19](#_Toc224506666)

[Figure S10 20](#_Toc224506667)

[Figure S11 21](#_Toc224506668)

[Figure S12 22](#_Toc224506669)

[Figure S13 23](#_Toc224506670)

[Figure S14 24](#_Toc224506671)

[Figure S15 25](#_Toc224506672)

[Figure S16 26](#_Toc224506673)

[Figure S17 28](#_Toc224506674)

[Figure S18 29](#_Toc224506675)

[Figure S19 30](#_Toc224506676)

[Figure S20 31](#_Toc224506677)

[Figure S21 32](#_Toc224506678)

[Figure S22 33](#_Toc224506679)

[Figure S23 34](#_Toc224506680)

[Figure S24 35](#_Toc224506681)

[Figure S25 36](#_Toc224506682)

[Figure S26 37](#_Toc224506683)

[Figure S27 38](#_Toc224506684)

[Figure S28 39](#_Toc224506685)

[Figure S29 40](#_Toc224506686)

[Figure S30 41](#_Toc224506687)

[Figure S31 43](#_Toc224506688)

[Supplementary Tables 45](#_Toc224506689)

[Table S1 45](#_Toc224506690)

[Table S2 46](#_Toc224506691)

[Table S3 47](#_Toc224506692)

[Table S4. 48](#_Toc224506693)

[Table S5. 49](#_Toc224506694)

[Table S6. 50](#_Toc224506695)

[References 52](#_Toc224506696)

# Experimental Section

## Chemicals

In this work, all chemicals were obtained from commercial sources and used without further purification. 1,3,5-triformylphloroglucinol (Tp, >97%) and 2,2′- bipyridine-5,5′-diamine (Bpy, >97 %) are provided by Shanghai Tensus Biotech CO., Ltd. N, N-dimethylacetamide (DMAc, 99.8%, with molecular sieves, water ≤50 ppm), 1, 4-dioxane (99 %), acetic acid (AcOH, ≥99.8 %) are from Aladdin. Molybdenum hexacarbonyl (Mo(CO)_6_, 98%) was provided by Macklin. Deionized water (18.2 MΩ, 300.6 K). Sonication (UC–240 40 KHZ) and centrifugation (H_2_-16K, 50 HZ) were used for re-dispersing and separation of the photocatalysts, respectively.

## Synthesis of Tp-Bpy COF

0.07 mmol Tp, and 0.12 mmol Bpy were added to a mixture of 2 mL DMAc and 1 mL 1, 4-dioxane into a 10 mL Pyrex tube. After the mixture was sonicated for 10 min, 0.3 mL AcOH (6 M) was added. Then, the Pyrex tube was degassed through three freeze-pump-thaw cycles, sealed off, and heated at 120°C for 72 h. The precipitate obtained by centrifugation was extensively washed with DMAc until the filtrate became colorless, followed by washing with THF, acetone, and dichloromethane. The resulting material was then dried under vacuum at 120°C for further use.

## Synthesis of Mo@Tp-Bpy COF

To prepare the Mo@Tp-Bpy COF samples, 40 mg of Tp-Bpy was dissolved in 20 mL of toluene and placed in a three-neck flask, sealed, and sonicated for 30 min to ensure complete dissolution. This solution was labeled as solution A. A toluene solution of Mo(CO)_6_ was then prepared with a concentration of 0.5 mg/mL, designated as solution B. Under a nitrogen atmosphere, varying volumes of solution B (2 mL, 11 mL, 22 mL, and 44 mL) were added dropwise to solution A, resulting in solution C. The resulting mixture was heated at 110°C for 6 hours under nitrogen protection, then cooled to room temperature and subjected to multiple washings with methanol and water. The solid product was then redispersed in 30 mL of toluene and irradiated with a 300 W xenon lamp under nitrogen for 20 minutes. Finally, the samples were vacuum-dried at 80°C for 12 hours, yielding the final powder products, labeled as Mo@Tp-Bpy. The samples were categorized based on the theoretical Mo content, corresponding to 1Mo@Tp-Bpy, 5Mo@Tp-Bpy (denoted as Mo@Tp-Bpy), 10Mo@Tp-Bpy, and 20Mo@Tp-Bpy. The actual Mo content was determined by inductively coupled plasma mass spectrometry (ICP).

## Characterization

X-ray diffraction (XRD) measurements were performed on a SmartLab (Rigaku) diffraction gauge with Cu Kα radiation at scanning rate of 2° min^-1^. A BRUKE Tensor-27 spectrometer was used for obtaining the Fourier transformation infrared spectroscopy (FT-IR), with a resolution of 4 cm^-1^. X-ray photoelectron spectroscopy (XPS) spectra were performed on a ESCALAB 250XI (Thermo system), the test results were corrected by taking C1s as 284.8 eV. The morphological characteristics of the composite sample were observed by JXA-840A (JEOL) field emission scanning electron microscopy (SEM), (TEM, JEOL JEM-2100) high-resolution transmission electron microscope (HR-TEM), along with energy dispersive X-ray spectroscopy (EDX) element mapping data. Aberration-corrected scanning transmission electron microscopy images were carried out by a JEOL 200F TEM operated at 200 keV, equipped with a probe spherical aberration corrector. Mo K-edge XAFS measurements was performed on the beamline BL01C1 in NSRRC. In order to investigate the light absorption ability of the samples, UV-visible diffuse reflectance absorbance tests were performed on the samples. The equipment used was an ultraviolet-visible photometer (UV-2600) from Shimadzu, Japan. The test conditions were: the substrate barium sulfate (BaSO_4_), and the test wavelength range was 200 nm - 1400 nm. Photoluminescence (PL) spectra and time-resolved photoluminescence (TRPL) were recorded on QuanmansterTM40 spectrophotometer (Photonics International inc.) and FLS1000 fluorescence lifetime spectrometer, respectively, with an excitation wavelength of 420 nm. In order to investigate the specific surface area and pore size distribution of the samples, the samples were tested for specific surface area (BET) using a fully automated specific surface and pore size distribution analyzer (Mac 2460). Prior to gas adsorption treatment samples were degassed at 150 ℃ for 12 h under vacuum. The hydrophilicity of the catalysts was investigated using a contact angle meter (DSA100, KRUSS, Germany) by dropping water droplets or 4-methoxybenzyl alcohol (4-MBA) solution (15.0 μL) on the surface of the samples. Transient-state surface photovoltage (TPV) response measurements were employed by a system equipped with a third-harmonic Nd: YAG laser (Polaris II, New Wave Research, Inc.) and a 500 MHz digital phosphor oscilloscope (TDS 5054, Tektronix).

## Photoelectrochemical Measurements

Photoelectrochemical measurements were performed using a CHI660E electrochemical analyzer (Shanghai Chenhua, China) in a standard three-electrode configuration. The prepared samples were deposited onto fluorine-doped tin oxide (FTO) glass substrates, which served as the working electrode, while a standard Ag/AgCl electrode and platinum foil were used as the reference and counter electrodes, respectively. A 0.5 mol L^-1^ Na_2_SO_4_ solution was used as the electrolyte. The specific steps of the working electrode of the photoelectric test sample are as follows: mix 250 μL ethanol, 250 μL ethylene glycol and 40 μL membrane solution (5% Nafion solution) into a mixed solution, then add 5 mg of the sample to be tested and disperse evenly by ultrasound. Apply the above solution evenly on conductive glass (make sure that the shape and area of coating on each glass are consistent), then bake at 60°C for 4 h. Transient photocurrent responses (i-t curves) were recorded under a 3 W LED lamp (365 nm) with periodic ON/OFF illumination cycles, using a 0.6 V bias potential. electrochemical impedance spectroscopy (EIS) measurements were conducted at a frequency range of 0.01–10^5^ Hz with an AC amplitude of 10 mV under open-circuit voltage conditions.

## Electron Paramagnetic Resonance (EPR)

The free radical signals were recorded using an EPR spectrometer (Bruker A300) with 5,5-dimethyl-1-pyrroline N-oxide (DMPO) as the trapping agent. The C-centered radical trapping test was performed under acetonitrile solution of DMPO with the addition of 4-MBA. 5 mg Ti-MOF@TB-COF was dispersed in an H_2_O/acetonitrile solution, and after purging with Ar for 30 min, 0.1 mL of 4-MBA and 100 μL of DMPO were added to the solution. The signals were collected under dark and light irradiation with a 420 nm LED.

## Femtosecond transient absorption (fs-TA) test

The fs-TA spectra for this study were obtained using a regenerative amplified Ti: sapphire laser system from Coherent (800 nm, 35 fs, 6 mJ pulse^-1^, 1 kHz repetition rate), nonlinear frequency mixing techniques, and the Femto-TA100 spectrometer (Time-Tech Spectra). The 800 nm output was split into two beams: one pumped a TOPAS Optical Parametric Amplifier (OPA) to generate the pump beam at 320 nm, while the other, after further splitting and attenuation (<10% of the original intensity) by a neutral density filter, was focused into a 2 mm thick sapphire window to generate a white light continuum (320-640 nm) serving as the probe beam. The pump fluence at the sample was maintained at 0.8 μJ cm^-2^ per pulse. The pump and probe beams were focused and overlapped onto the sample using Al parabolic reflectors. The sample was prepared as an aqueous dispersion (0.5 mg mL^-1^). After passing through the sample, the probe beam was collimated and directed into a fiber-coupled spectrometer with CMOS sensors, detected at a 1 kHz frequency. The pump-probe delay was controlled using a motorized delay stage. The pump pulses were chopped at 500 Hz by a synchronized chopper, and the absorbance change was calculated by comparing two adjacent probe pulses (pump-blocked and pump-unblocked).

## In situ diffuse reflectance infrared Fourier transform spectroscopy (In situ DRIFTS) test

The photocatalyst with addition of 4-MBA solution was placed in the cuvette and kept under CO_2_ atmosphere for 30 min. The infrared spectra were recorded on a diffuse reflectance infrared Fourier transform spectrometer (Nicolet iS10, Thermo). The infrared spectra were recorded from 0 to 20 min during irradiation with a 420 nm LED lamb (3W).

## Photocatalytic CO_2_ reduction and 4-methoxybenzyl alcohol (4-MBA) oxidation measurement

The CO_2_ photoreduction to CO and the concurrent oxidation of 4-MBA to AA were carried out in a 25 mL quartz reactor. 5 mg of catalyst, 9 mL of H_2_O, and 1 mL of 4-MBA were added to the reactor. Subsequently, high-purity CO_2_ (99.999%) was introduced into the reactor for 30 min. The dispersion was then stirred uniformly at 300 rpm at 25°C and 1 atm CO_2_ pressure. Simulated light irradiation was provided by a 300 W Xe lamp (CEL-PE300L-3A). The gaseous products, H_2_ and CO, were quantitatively analyzed using a gas chromatograph (GC-2014, Japan, Shimadzu) equipped with a thermal conductivity detector (TCD) and a flame ionization detector (FID). After 3 h of reaction, the liquid product was collected and analyzed using an Agilent A91PLUS gas chromatograph. The heating program was as follows: initial temperature held at 85°C for 2 min, increased to 210°C at a rate of 10°C/min, then raised to 220°C at a rate of 5°C/min. Helium was used as the carrier gas at a flow rate of 1 mL/min. For recycling experiments, the reactor was purged with CO_2_ for 30 min after each cycle. The selectivity of CO was calculated using the following formula:

$$\text{Selectivity (\%) =}\frac{\text{C}\text{CO}}{\text{C}\text{CO}\text{+}\text{C}\text{H2}}\text{ × 100\%}$$

The apparent quantum efficiency (AQE) for CO_2_ reduction and 4-MBA oxidation generation is calculated according to the following equation:

$$\text{AQE=}\frac{\text{Numbers of evolved }\text{CO+}\text{H}\text{2}\text{+}\text{AA}}{\text{Numbers of incident photons}}\text{ × 100\%}$$

$$\text{=}\frac{\text{M}\text{×}\text{N}\text{A}\text{×}\text{c}\text{×}\text{h}}{\text{p}\text{×}\text{t}\text{×}\text{λ}}\text{ × 100\%}$$

Where M represents the amount of produced CO+H_2_+AA, N_A_ is Avogadro constant, c is the speed of light, h is the Planck constant, P is the intensity of the irradiation, t is the time of illumination, and λ is the wavelength.

## DFT calculation details

The Vienna ab initio simulation package (VASP, version 5.4.4) ^[1]^ was utilized to calculate the properties of catalysts. Core electrons were described with projector augmented wave (PAW) pseudopotentials, and valence electrons were represented by plane waves with a kinetic energy cut-off of 450 eV.^[2, 3]^ The Perdew-Burke-Ernzerhof (PBE) generalized gradient approximation (GGA) exchange-correlation functional was applied,^[4]^ and the DFT-D3 method was used for dispersion corrections.^[5]^ Geometry optimizations were performed using a 1×1×1 k-point mesh, with energy and force convergence set to 1x10^-5^ eV and 0.05 eV/Å, respectively.The reaction energy profiles were determined using the computational hydrogen electrode model,^[6]^ for which the Gibbs free energy change of each step was defined as

∆G = ∆E + ∆E_ZPE_ – T∆S

where ∆E_ZPE_ is the difference in zero-point energy, and ∆S is the difference in entropy.^[7]^

## The equations for calculating bandgap, HOMO and LUMO positions

Equation 1

(αhv)^1/n^ = A(hv - E_g_) (1)

where α is the absorbance coefficient, h is Planck's constant, v is frequency, E_g_ is the band gap of the semiconductor, and A is constant. where n is related to the semiconductor type, n is 1/2 for direct bandgap semiconductors and 2 for indirect bandgap semiconductors.

Equation 2

E_LUMO_=E_HOMO_-E_g_ (2)

E_LUMO_ is the conduction band value, E_HOMO_ is the valence band value, and E_g_ is the band gap.

# Supplementary Figures





Figure S1. Optimized surface structures of the Tp-Bpy COF with various interlayer metal atoms incorporated.





Figure S2. Optimized CO_2_ adsorption configurations of Tp‑Bpy COF with different interlayer metal atoms.


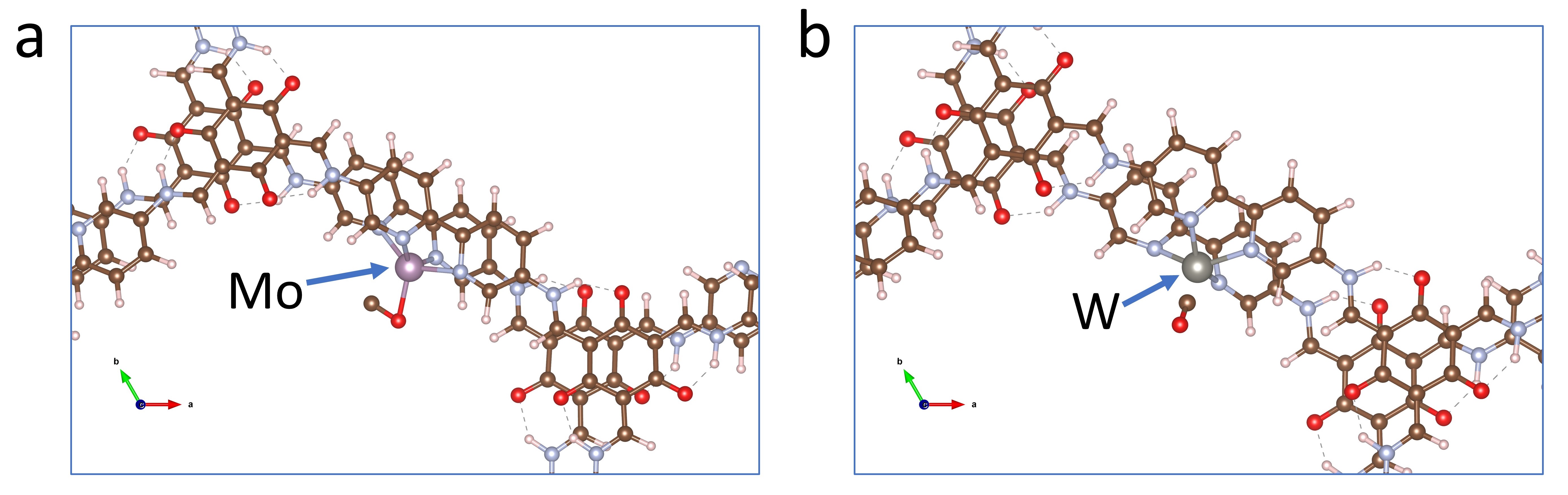


Figure S3. Optimized CO_2_ adsorption configurations of Tp‑Bpy COF with (a) Mo and (b) W.


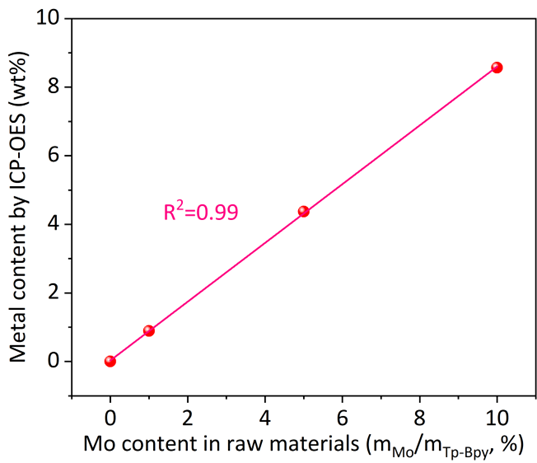


Figure S4. ICP-OES results of samples loaded with different Mo content.


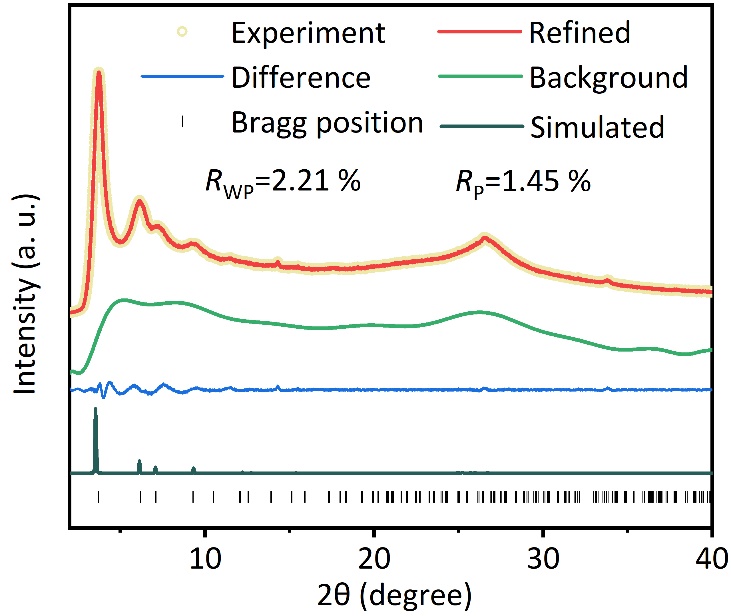


Figure S5. XRD pattern of pristine Tp-Bpy.

Note: **Figure S5** presents the Rietveld refinement results of the powder X-ray diffraction (XRD) data for the crystalline Tp-Bpy COF material. The excellent agreement between the experimental pattern and the refined profile, along with the nearly flat difference curve and low reliability factors (R_WP_ = 2.21%, R_P_ = 1.45%), collectively validates the accuracy of the structural model. The theoretical diffraction pattern confirms an AA stacking mode.


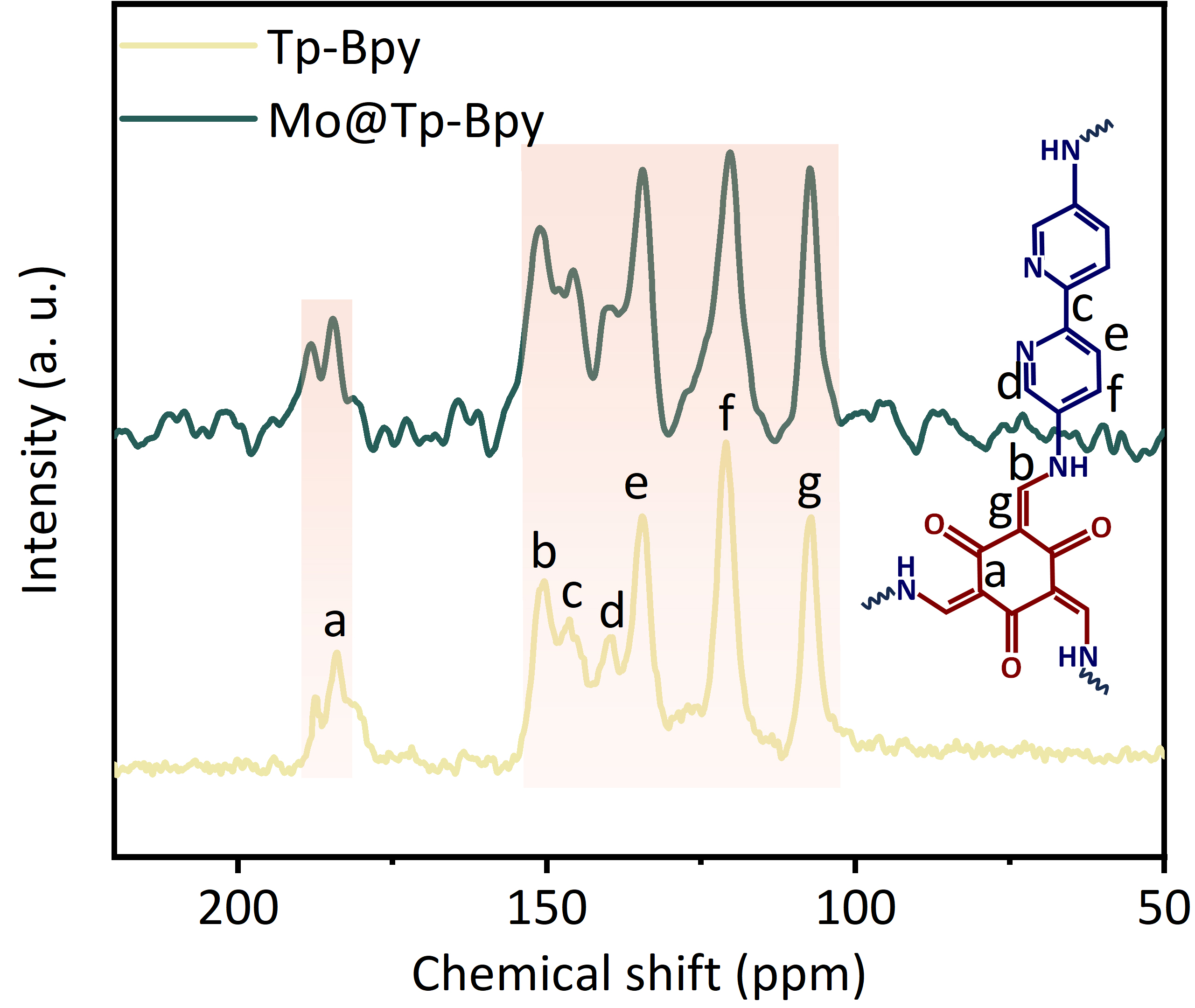


Figure S6. Solid-state ^13^C NMR spectra of Tp-Bpy and Mo@Tp-Bpy.

Note: Tp-Bpy and Mo@Tp-Bpy display characteristic signals corresponding to aromatic carbons of Tp and Bpy units, as well as imine-linker carbons (denoted as a-g).


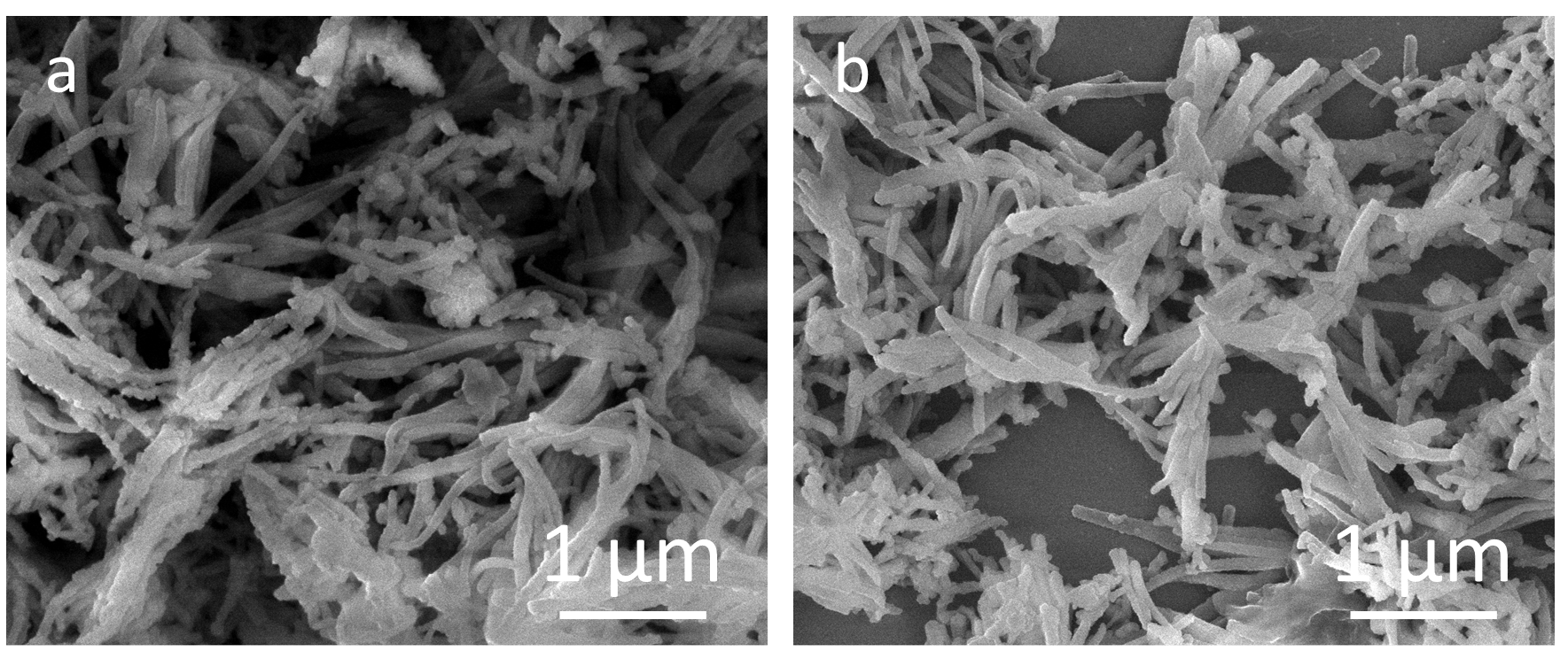


Figure S7. SEM images of (a) Tp-Bpy and (b) Mo@Tp-Bpy.


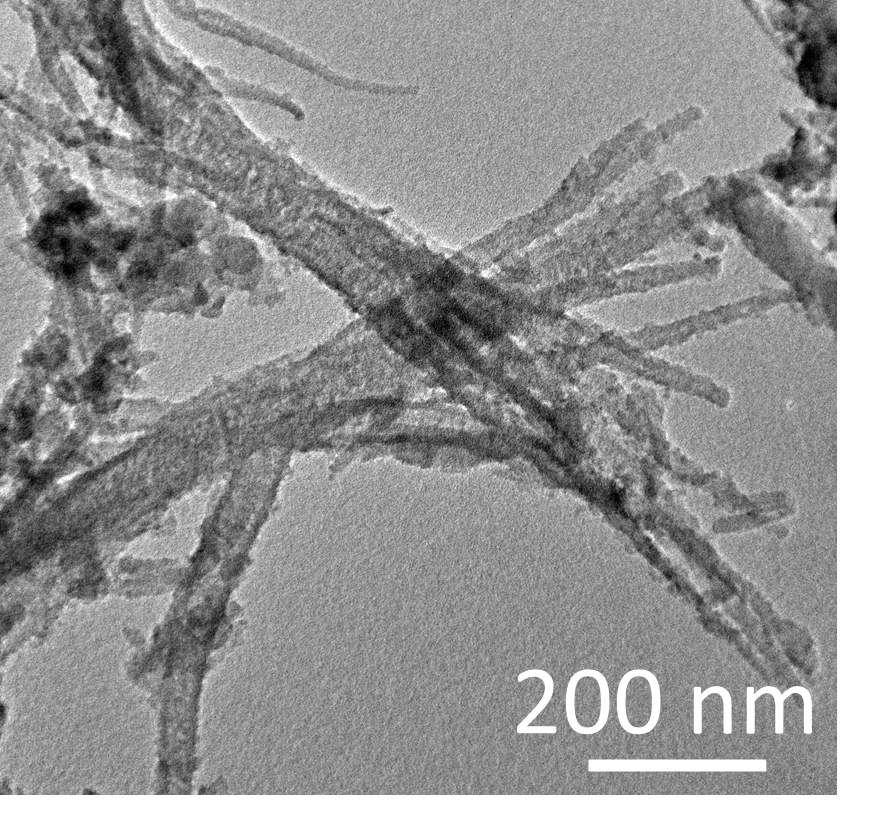


Figure S8. TEM image of pristine Tp-Bpy.


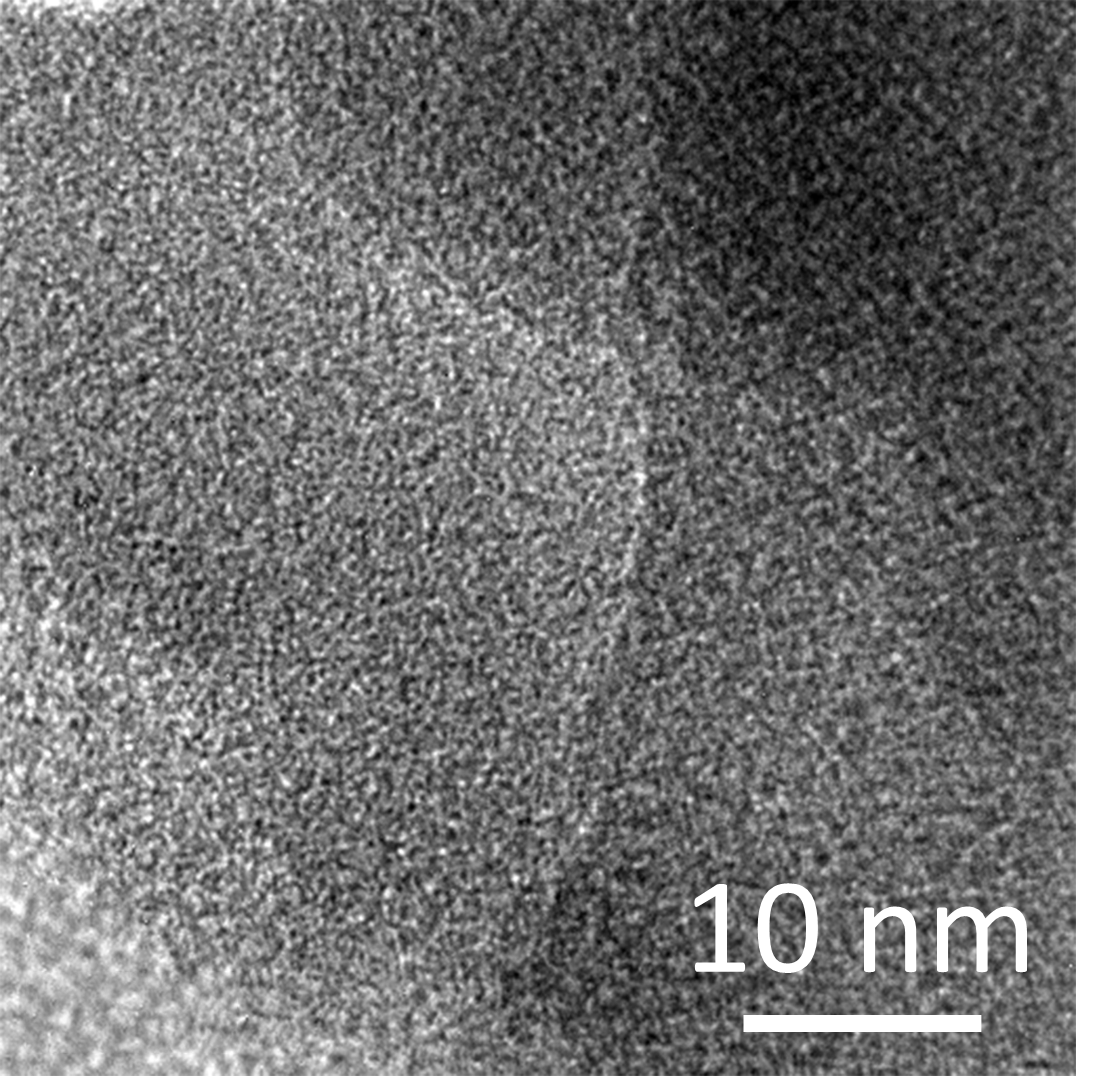


Figure S9. HR-TEM image of Mo@Tp-Bpy.


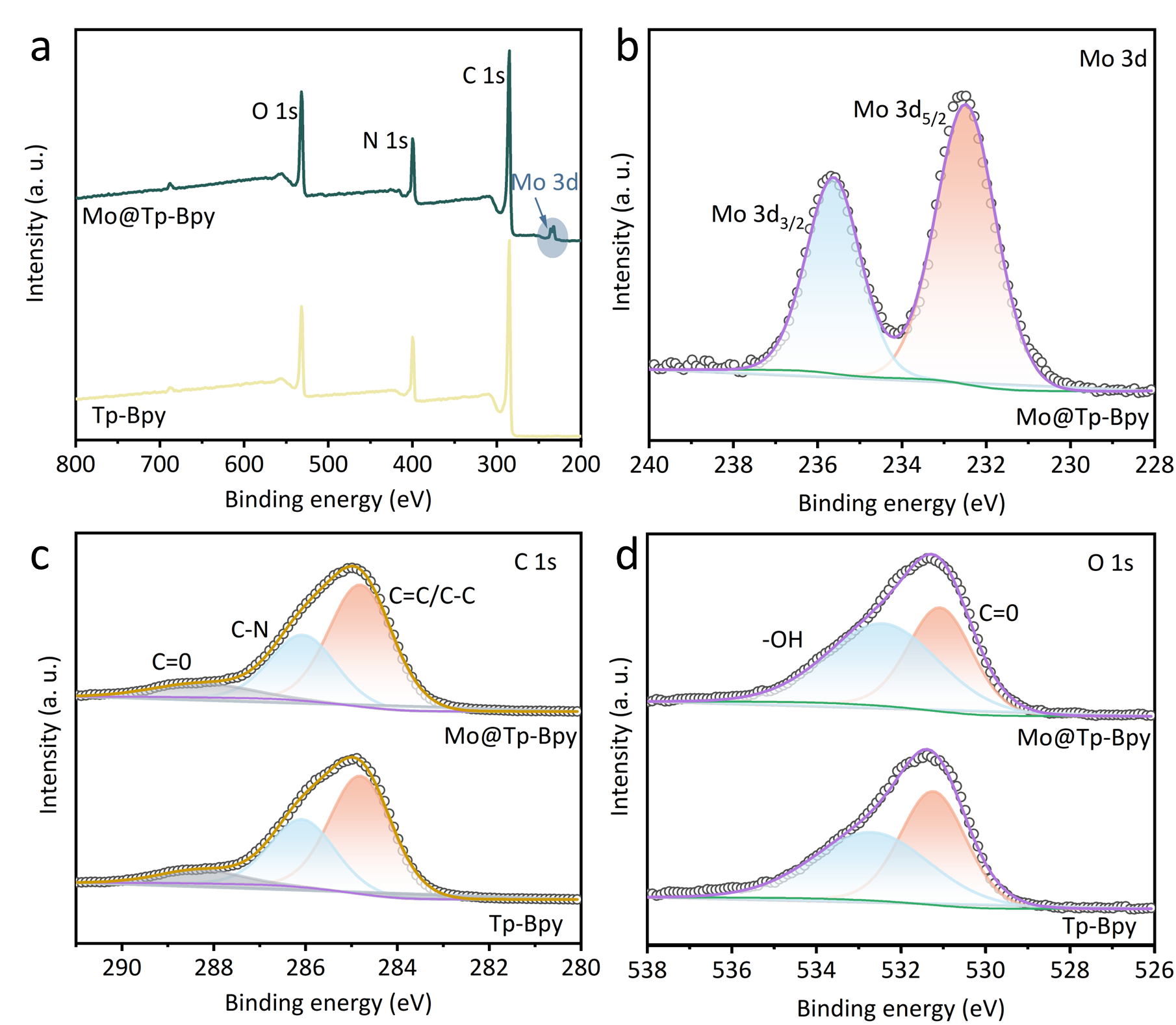


Figure S10. XPS spectra of (a) survey, (b) Mo 3d, (c) C 1s and (d) O 1s of Tp-Bpy and Mo@Tp-Bpy.

Note: Survey scans (**Figure S10a**) confirm the presence of C, N, O, and Mo in Mo@Tp-Bpy. The Mo 3d spectrum (**Figure S10b**) exhibits doublet peaks at 232.5 eV (3d_5/2_) and 235.6 eV (3d_3/2_), indicative of Mo in the +6-oxidation state. The C 1s spectra (**Figure S10c**) of Tp-Bpy and Mo@Tp-Bpy both show three contributions at 284.8 eV (C=C), 286.1 eV (C–N), and 288.3 eV (C=O), with no significant changes upon Mo loading, suggesting that the carbon-based chemical environment and conjugated structure of the framework remain intact. This further supports that Mo anchoring occurs primarily through N sites rather than Csites. The O 1s spectra (**Figure S10d**) display two peaks at 531.2 eV (C=O) and 532.7 eV (adsorbed H_2_O or –OH), consistent with the expected chemical environment.


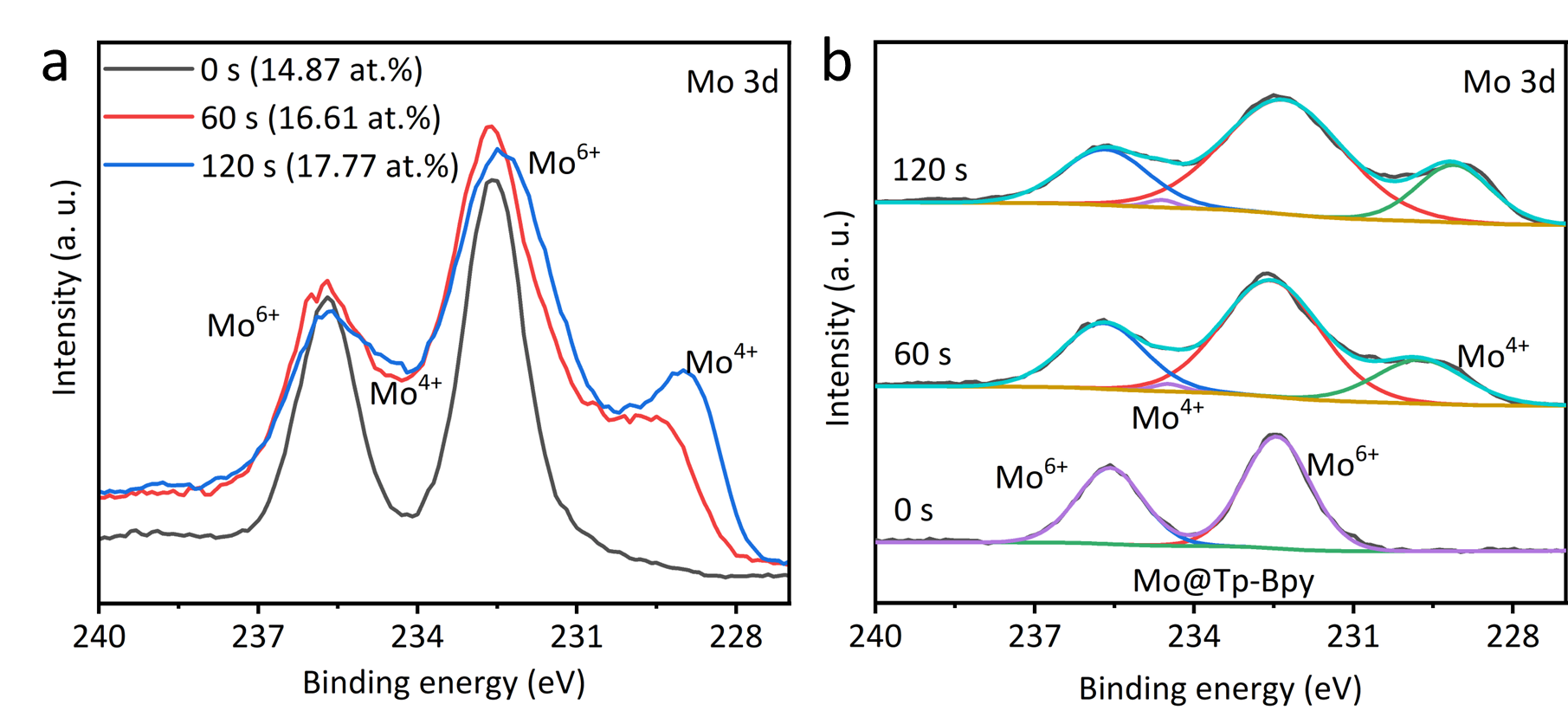


Figure S11. The Mo 3d XPS spectra collected before Ar^+^ sputtering (0 s) and at different times after sputtering (60 s and 120 s).

Note: To gain insight into the bulk composition, depth profiling was conducted via gradual Ar^+^ sputtering to remove the surface layers. The Mo content was observed to increase from 14.87 at% to 17.77 at% with prolonged sputtering time (**Figure S11** and **Table S2**), indicating a concentration gradient from the surface region to the bulk. This finding confirms that the majority of Mo atoms are incorporated within the interlayers of Tp-Bpy. Concurrently, due to bombardment by high-energy ion beams, some of the highly oxidized +6-valent Mo is reduced to the lower-valent +4-valent Mo. [8]


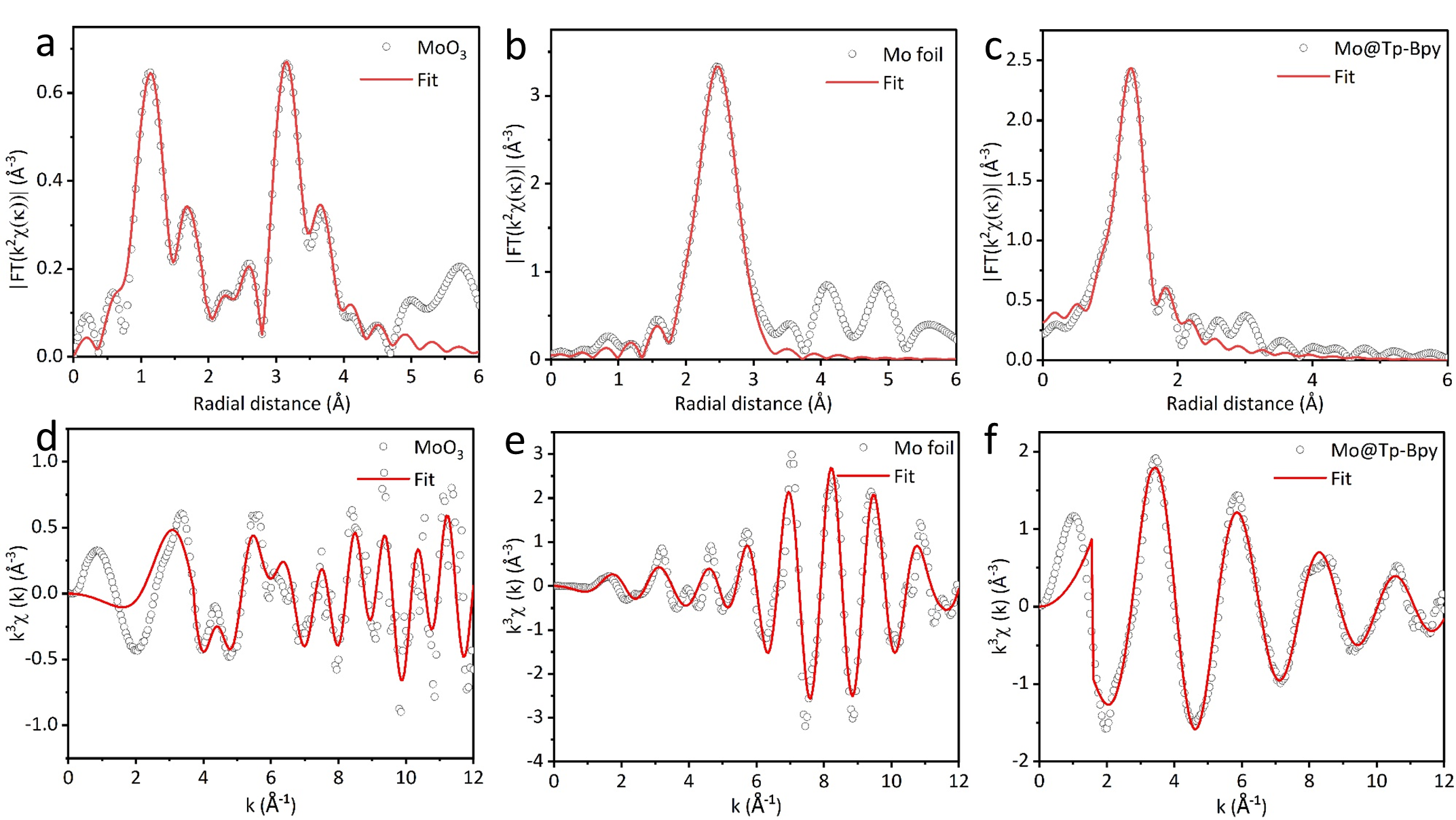


Figure S12. R-space EXAFS fitting plots of (a) MoO_3_, (b) Mo foil and (c) Mo@Tp-Bpy. k^3^-weighted EXAFS fitting curves of (d) MoO_3_, (e) Mo foil and (f) Mo@Tp-Bpy.


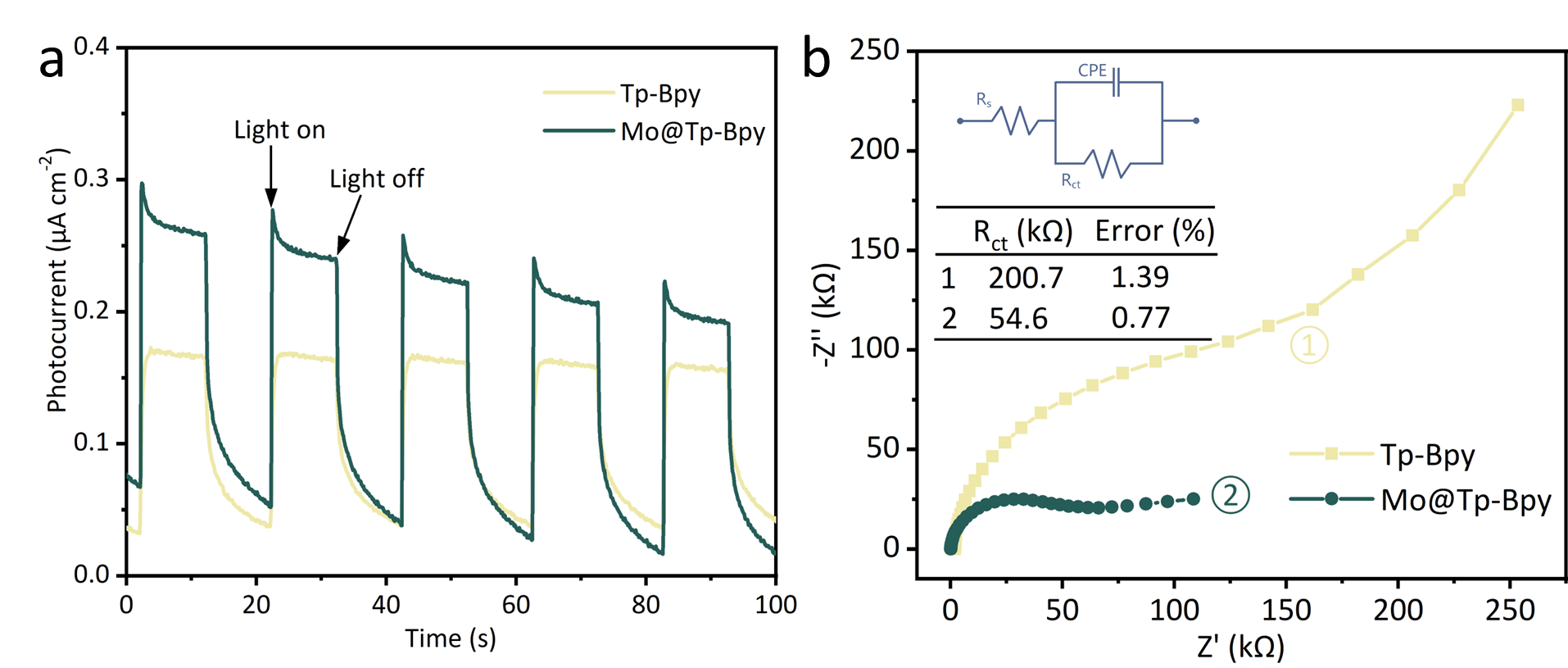


Figure S13. (a) Transient photocurrent response and (b) EIS Nyquist plots of different sapmles.

Note: Under periodic light irradiation, Mo@Tp-Bpy exhibits a significantly enhanced photocurrent response compared to Tp-Bpy (**Figure S13a**), indicating markedly improved charge separation and transfer efficiency upon the introduction of Mo single atoms. Electrochemical impedance spectroscopy (EIS) further supports this conclusion. The Nyquist plots fitted with an equivalent circuit model reveal a substantial decrease in the charge transfer resistance (Rct), from 200.7 kΩ for Tp-Bpy to only 54.6 kΩ for Mo@Tp-Bpy, with minimal fitting error (**Figure S13b**). This pronounced reduction in interfacial resistance demonstrates that the atomically dispersed Mo species serve as effective charge-transfer mediators, thereby optimizing the interlayer charge migration process and suppressing bulk recombination.


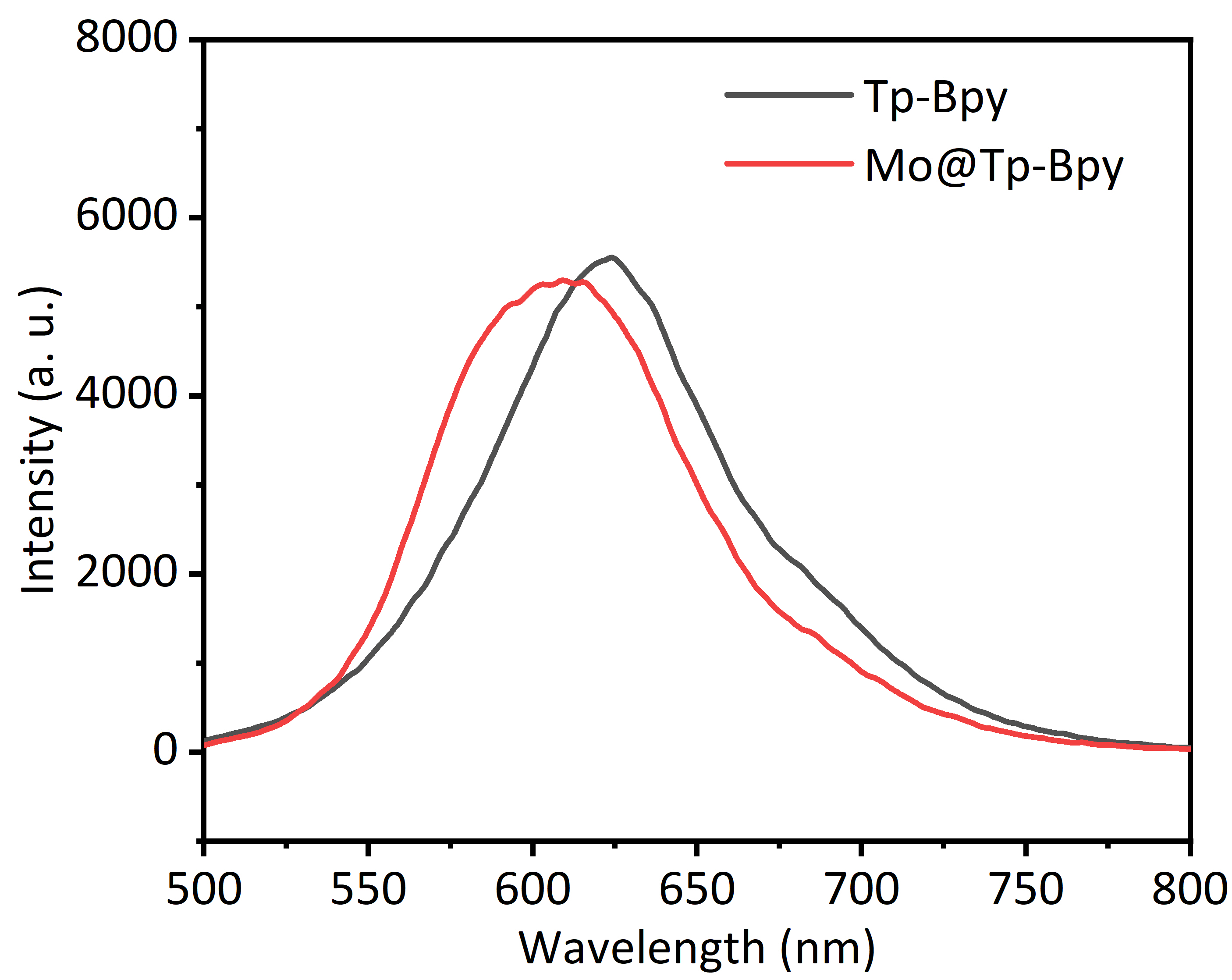


Figure S14. PL spectra of different samples.

Note: Mo@Tp-Bpy exhibits a significantly quenched emission intensity, particularly in the 550-700 nm region corresponding to the π-π* transition of the framework. This observation clearly indicates that Mo single atoms effectively capture photogenerated electrons, thereby suppressing charge carrier recombination.


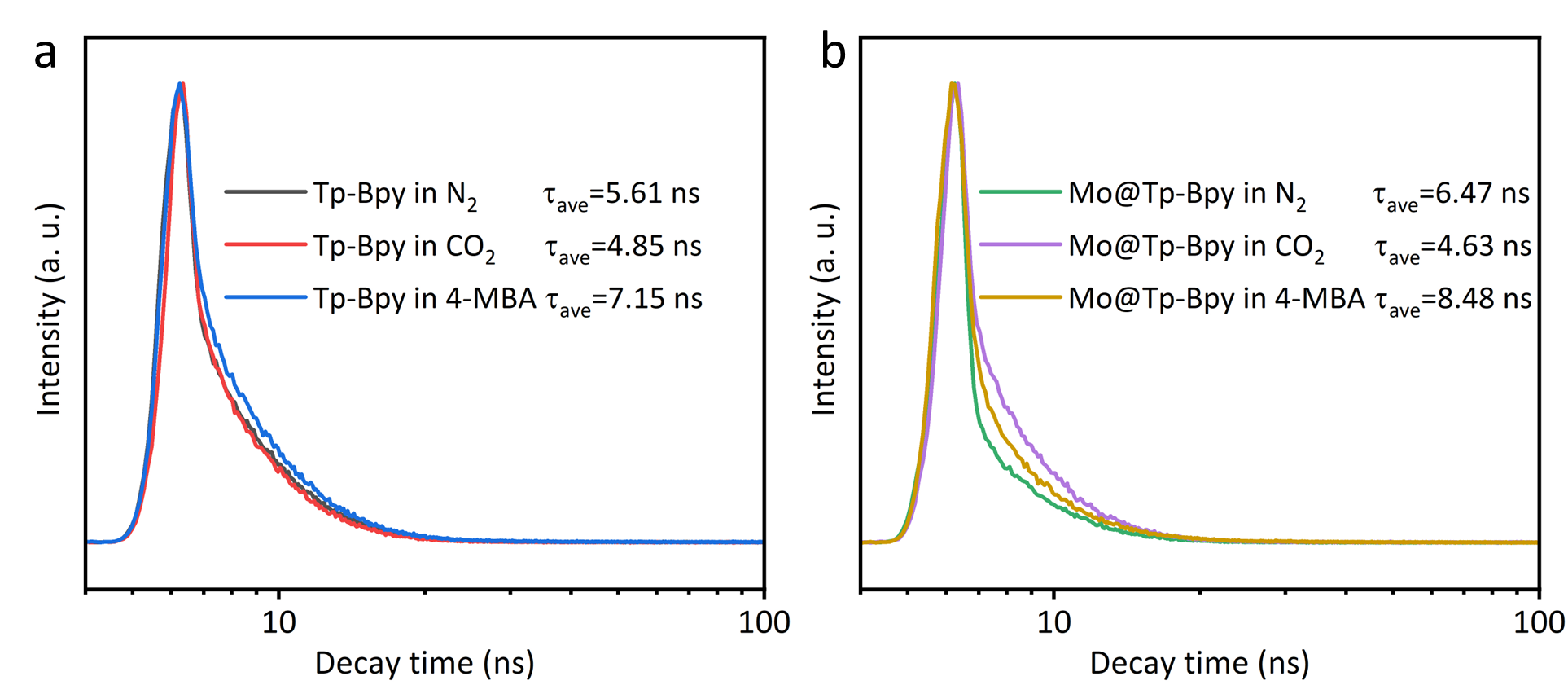


Figure S15. TR-PL decay curves of (a) Tp-Bpy and (b) Mo@Tp-Bpy in different conditions.

Note: TR-PL decay experiments conducted under varying conditions further revealed the carrier dynamics and reactant response behaviors. Evidently, under a N_2_ atmosphere, the average photoluminescence lifetime of Mo@Tp-Bpy (6.47 ns) was significantly longer than that of Tp-Bpy (5.61 ns), consistent with electron transport induced by Mo and suppressed non-radiative recombination. Upon switching to a CO_2_ atmosphere, the lifetimes of both catalysts decreased, indicating that CO_2_ molecules act as electron acceptors, capturing photogenerated electrons. With Mo@Tp-Bpy exhibiting a more pronounced drop from 6.47 ns to 4.63 ns, which highlights the critical role of the exciton confinement effect of Mo atoms in enhancing CO_2_ adsorption and activation, thereby facilitating efficient electron transfer for CO_2_ reduction. On the other hand, when samples were subjected to 4-MBA conditions, the average lifetime significantly increased, primarily due to the effective consumption of photogenerated holes by 4-MBA, which enhanced the generation of excited-state electrons.


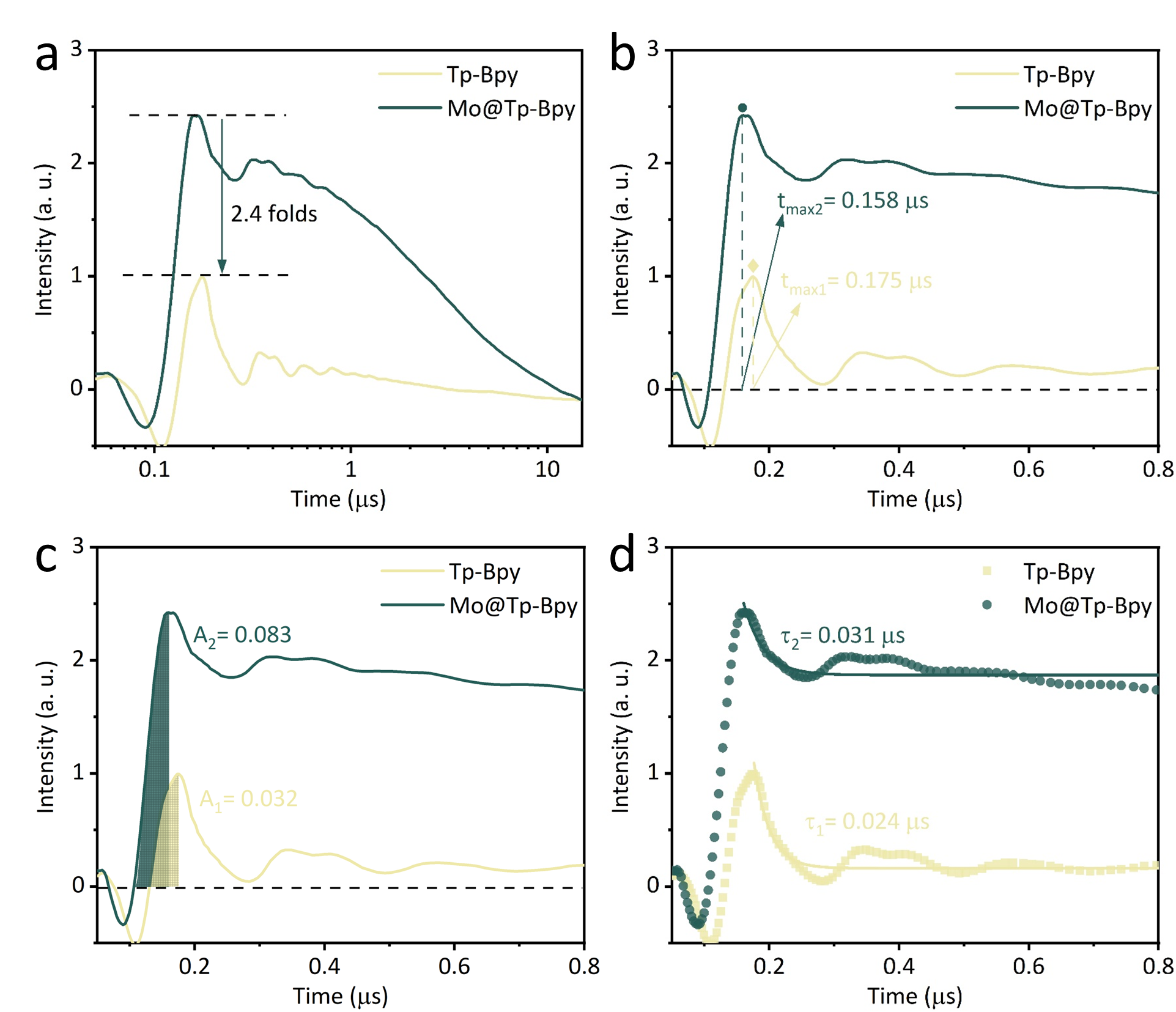


Figure S16. (a) TPV intensity, (b) maximum charge extraction time (*t_max_*), (c) amount of charge extraction, and (d) attenuation constants (*τ*) of different samples.

Note: Transient photovoltage (TPV) measurements were performed. Key kinetic parameters including the maximum charge extraction time (t_max_, reflecting electron extraction rate/diffusivity), the extracted charge quantity (A, represented by the integrated area under the TPV decay curve), and the interfacial charge lifetime (τ) were systematically analyzed to provide a comprehensive understanding of charge separation. As shown in **Figure S16a**, Mo@Tp-Bpy exhibits a photovoltage signal intensity 2.4 times higher than that of Tp-Bpy, indicating significantly enhanced charge separation efficiency. Moreover, Mo@Tp-Bpy shows a shorter t_max_ value (0.158 μs) compared to Tp-Bpy (0.175 μs, **Figure S16b**), suggesting faster electron extraction driven by the Mo single-atom sites. Notably, the integrated signal area A for Mo@Tp-Bpy (0.083) is 2.6 times larger than that of Tp-Bpy (0.032, **Figure S16c**), confirming a greater number of photogenerated charges reaching the catalyst surface per unit time. In addition, Mo@Tp-Bpy exhibits a prolonged charge lifetime (τ_2_ = 0.031 μs) relative to Tp-Bpy (τ_1_ = 0.024 μs, **Figure S16d**), which can be attributed to the role of Mo sites as efficient electron traps that retard electron–hole recombination, coupled with enhanced electron delocalization that optimizes the interfacial electric field. The effective surface charge density (A_eff_), calculated via Equation $A_{eff}=A\tau/t_{max}$, further highlights the superiority of Mo@Tp-Bpy, with a value of 0.016 compared to 0.004 for Tp-Bpy. This enhancement stems from the AIETB established by the Mo–N_4_ sites, which promote ultrafast electron migration.


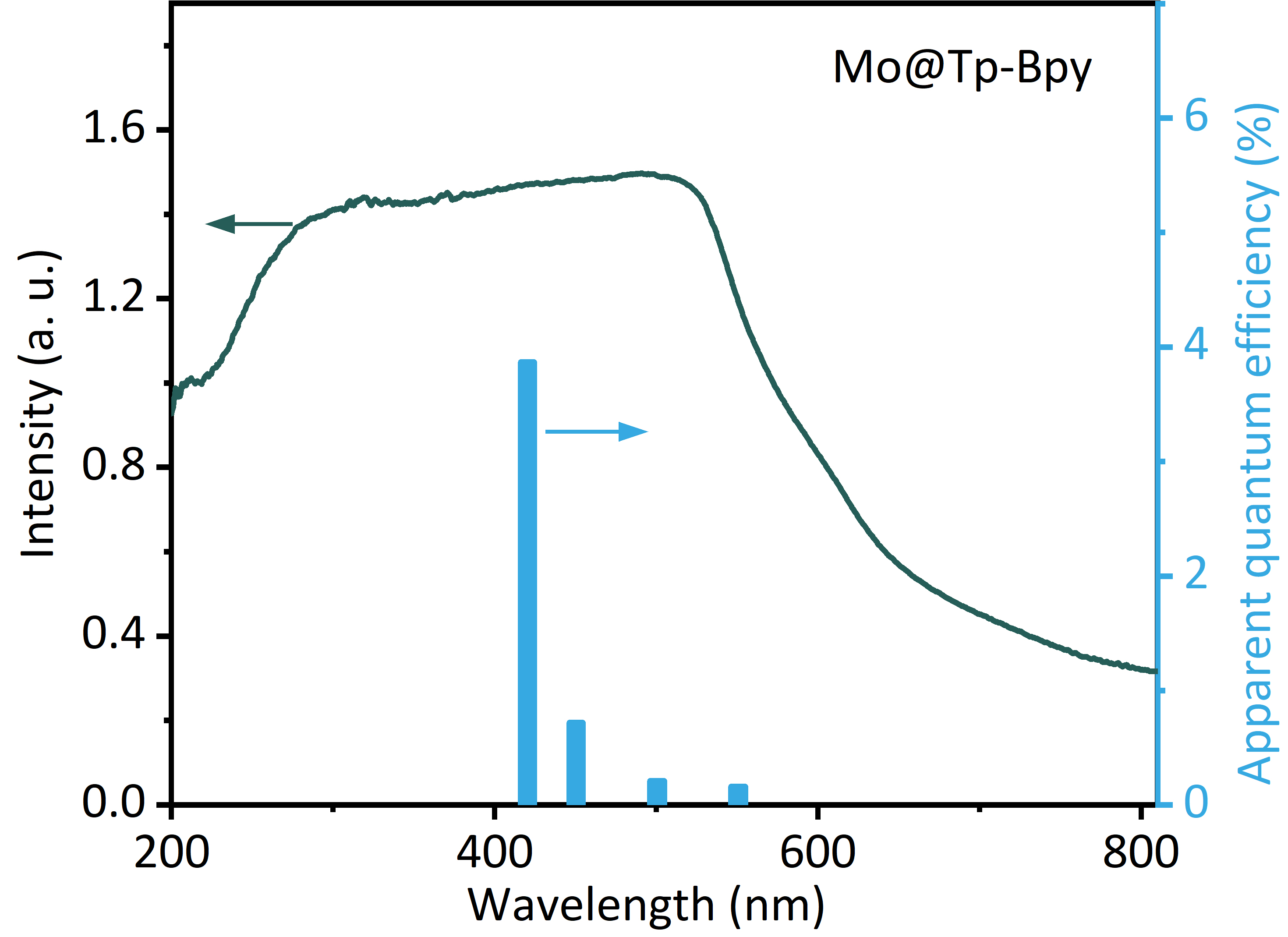


Figure S17. AQE and UV-vis absorption of Mo@Tp-Bpy.


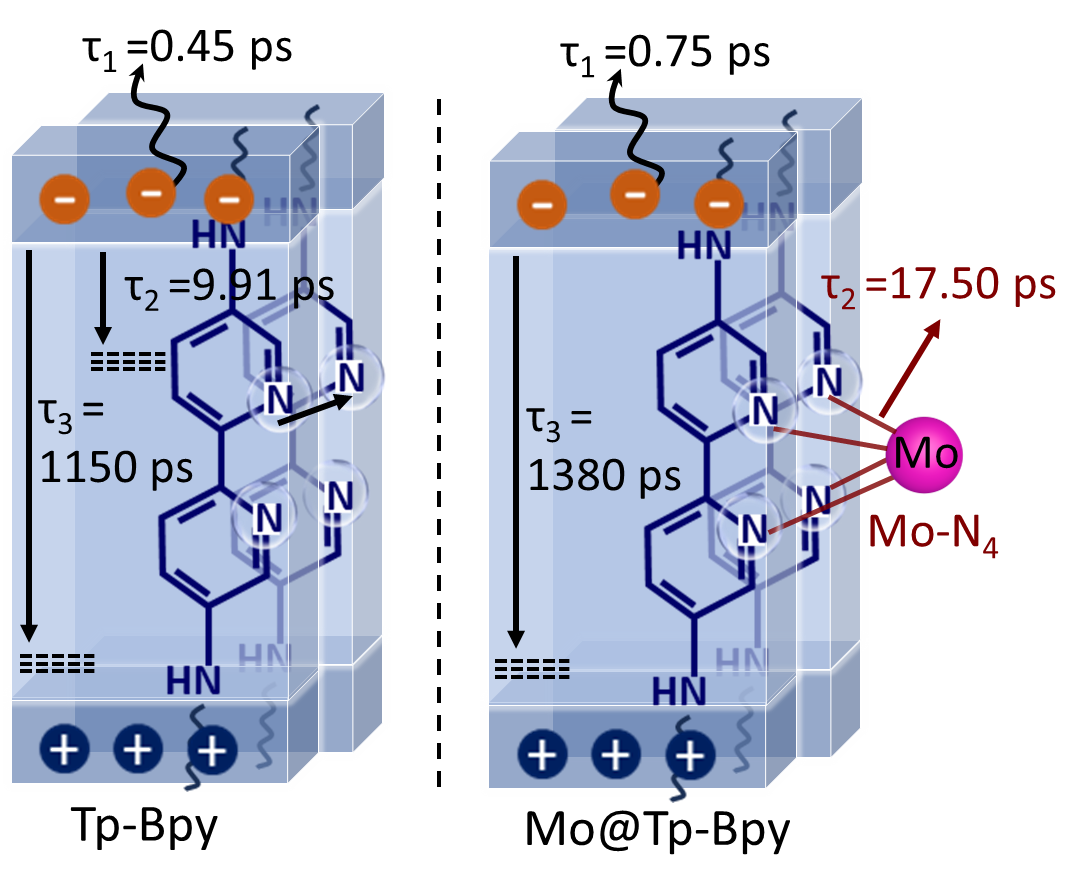


Figure S18. Schematic illustration of excited-state dynamics for Tp-Bpy and Mo@Tp-Bpy.


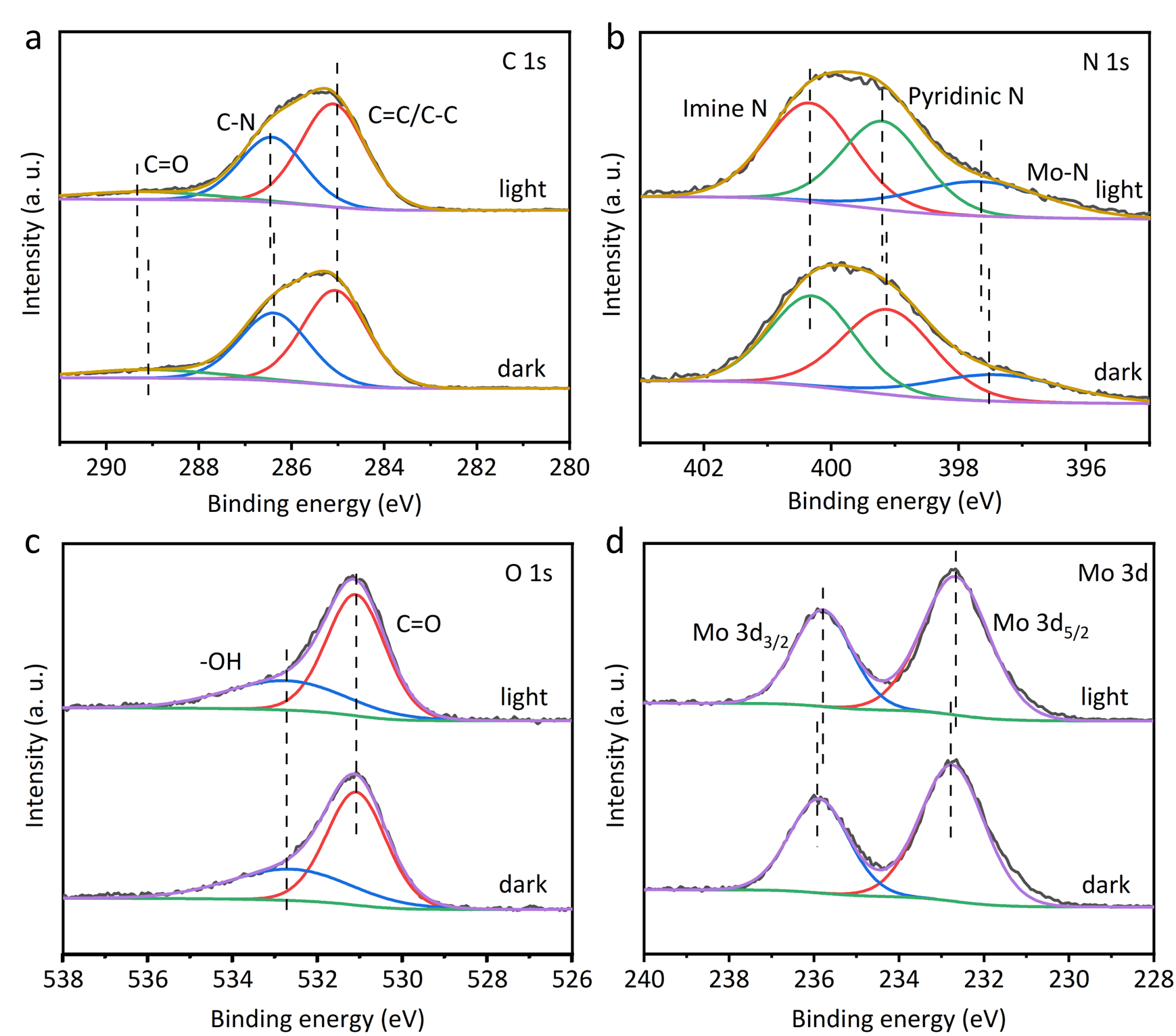


Figure S19. ISI-XPS spectra of (a) C, (b) N, (c) O and (d) Mo.

Note: In situ irradiation X-ray photoelectron spectroscopy (ISI-XPS) was employed to track the electron transfer pathways under operating conditions. Upon visible light irradiation, the Mo 3d peaks shifted toward lower binding energy by approximately 0.12 eV, while the C 1s, N 1s, and O 1s peaks exhibited positive shifts. This opposite shift direction demonstrates that photogenerated electrons are transferred from the photoexcited Tp-Bpy COF framework to the Mo centers via the Mo–N bonds. This result is consistent with the fs-TAS and calculated PDOS profiles, indicating that the interlayer Mo SAs introduce mid-gap states within the Tp-Bpy COF, providing additional electron pathways for charge transfer.


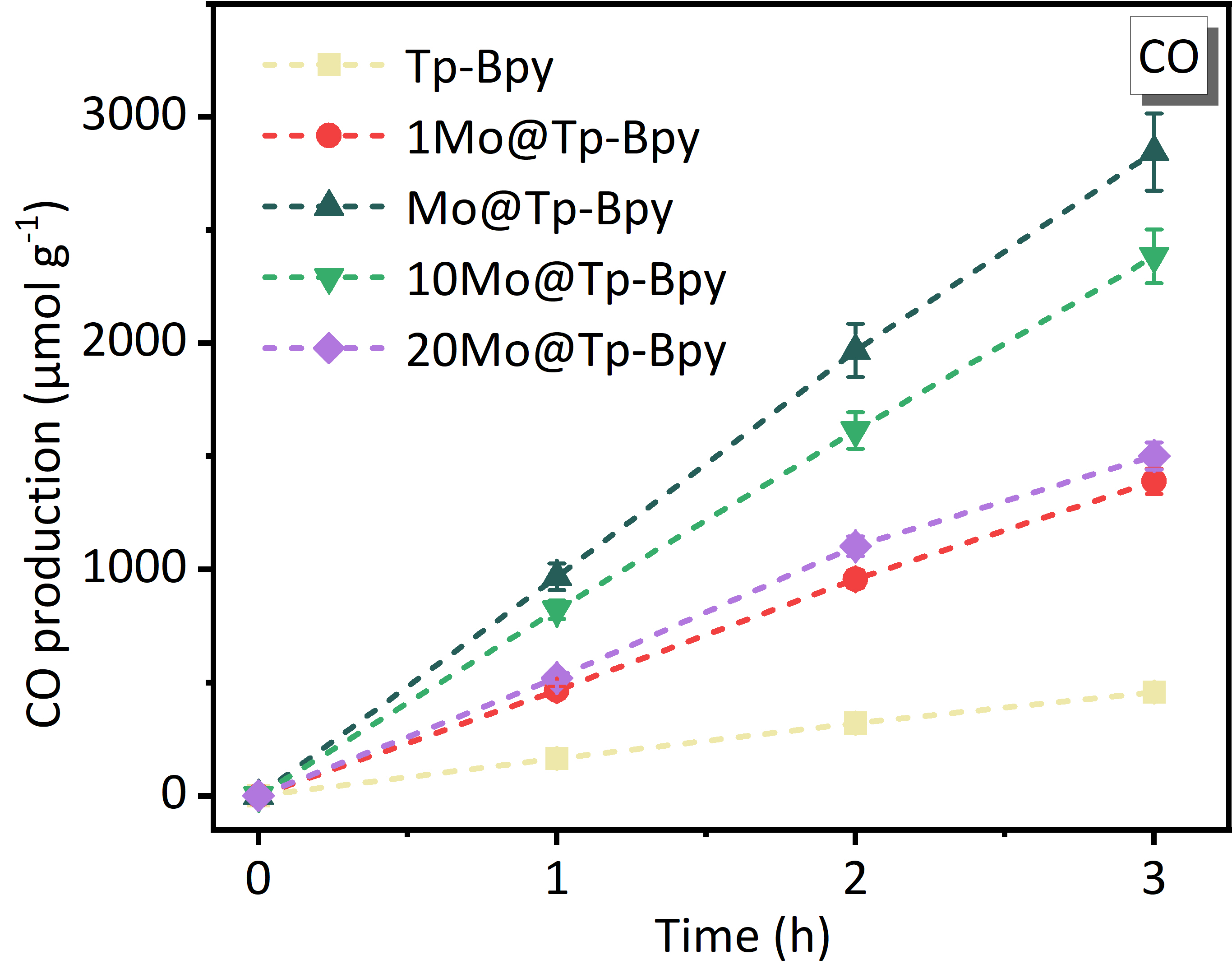


Figure S20. Time-dependent CO production in CO_2_ photoreduction over Tp-Bpy and Mo@Tp-Bpy with different Mo loadings.


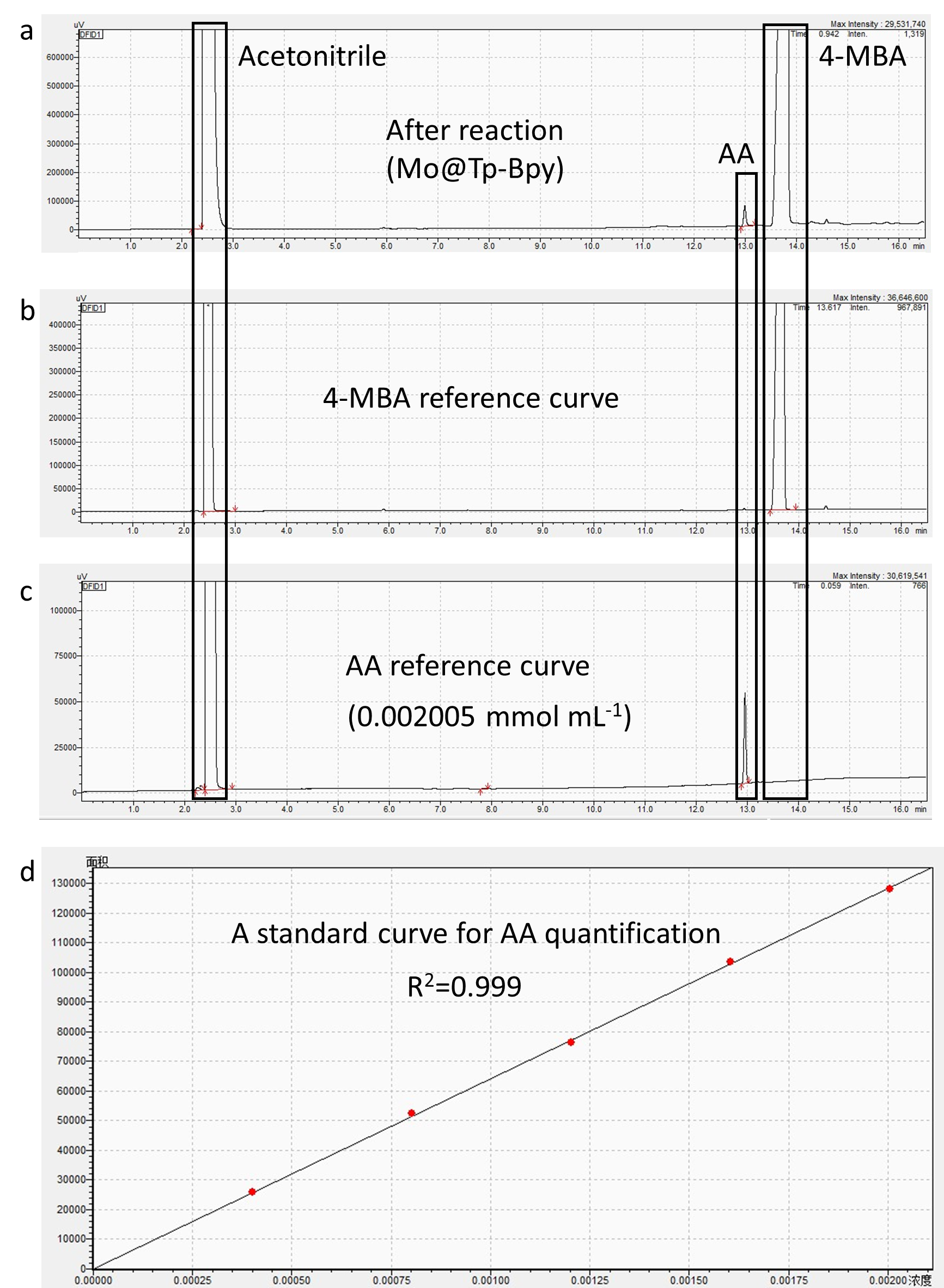


Figure S21. The quantitative calibration curve for the reacted products.


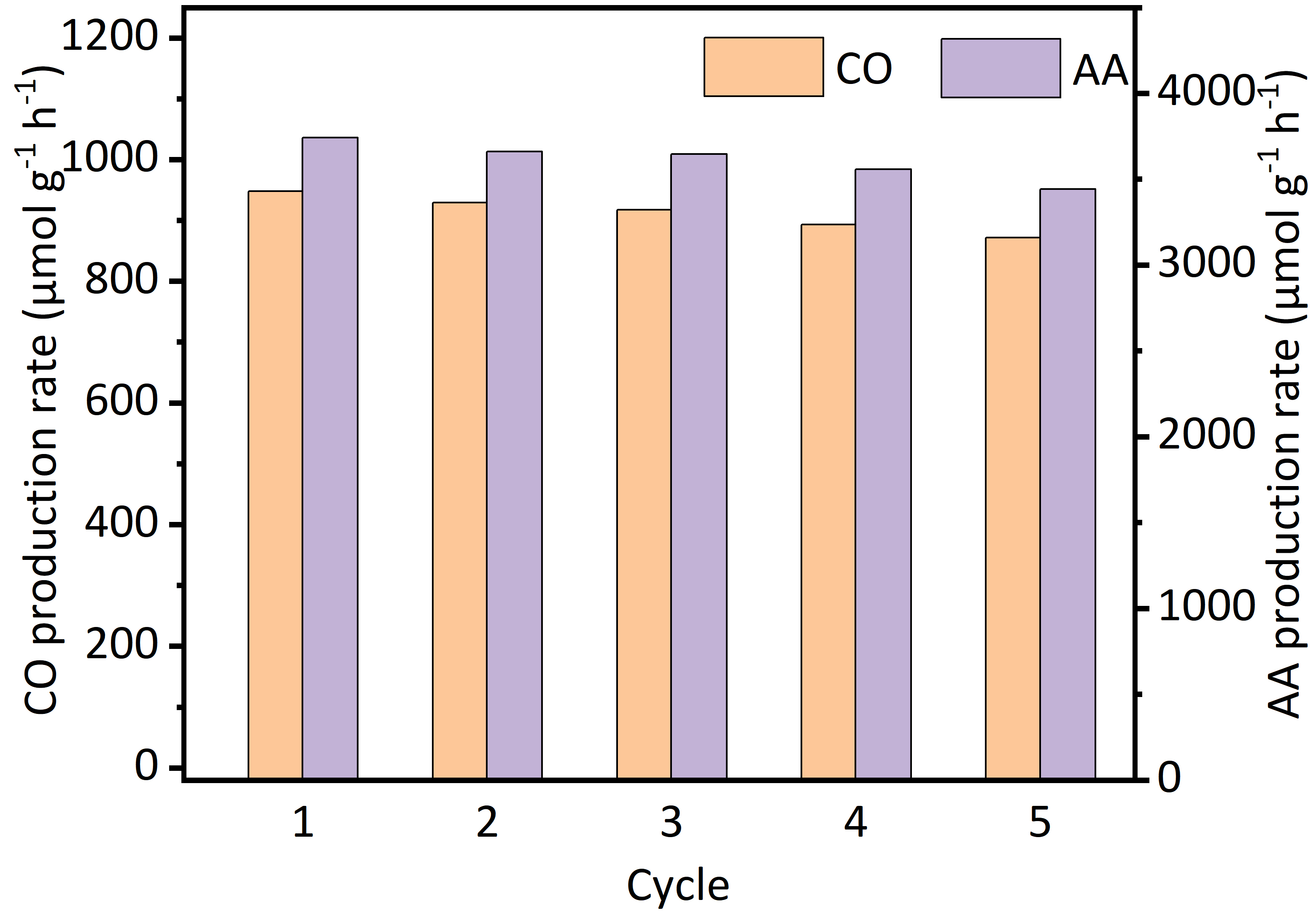


Figure S22. Cycling stability test of Mo@Tp-Bpy for simultaneous CO_2_ reduction and 4-MBA oxidation.


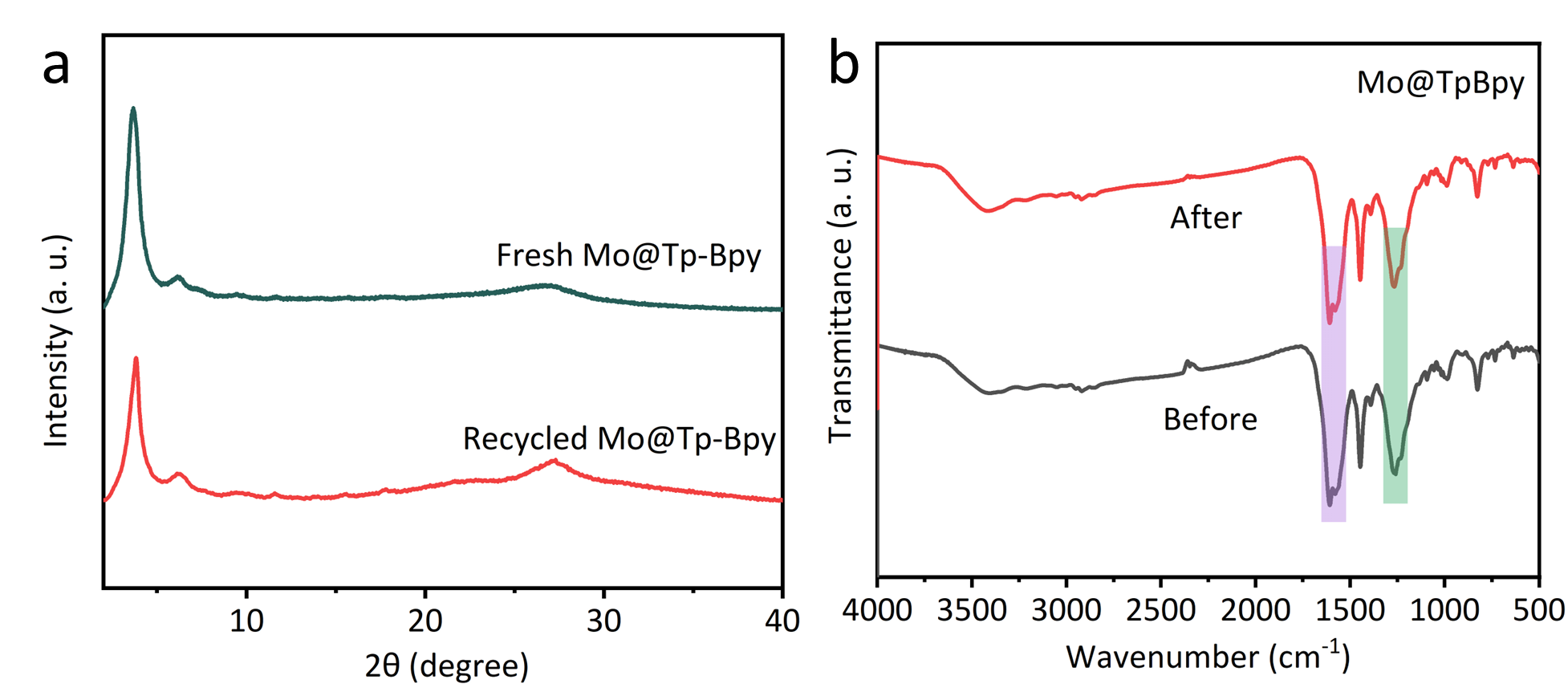


Figure S23. (a) XRD patterns and (b) FTIR spectra for Mo@Tp-Bpy before/after the durability test.


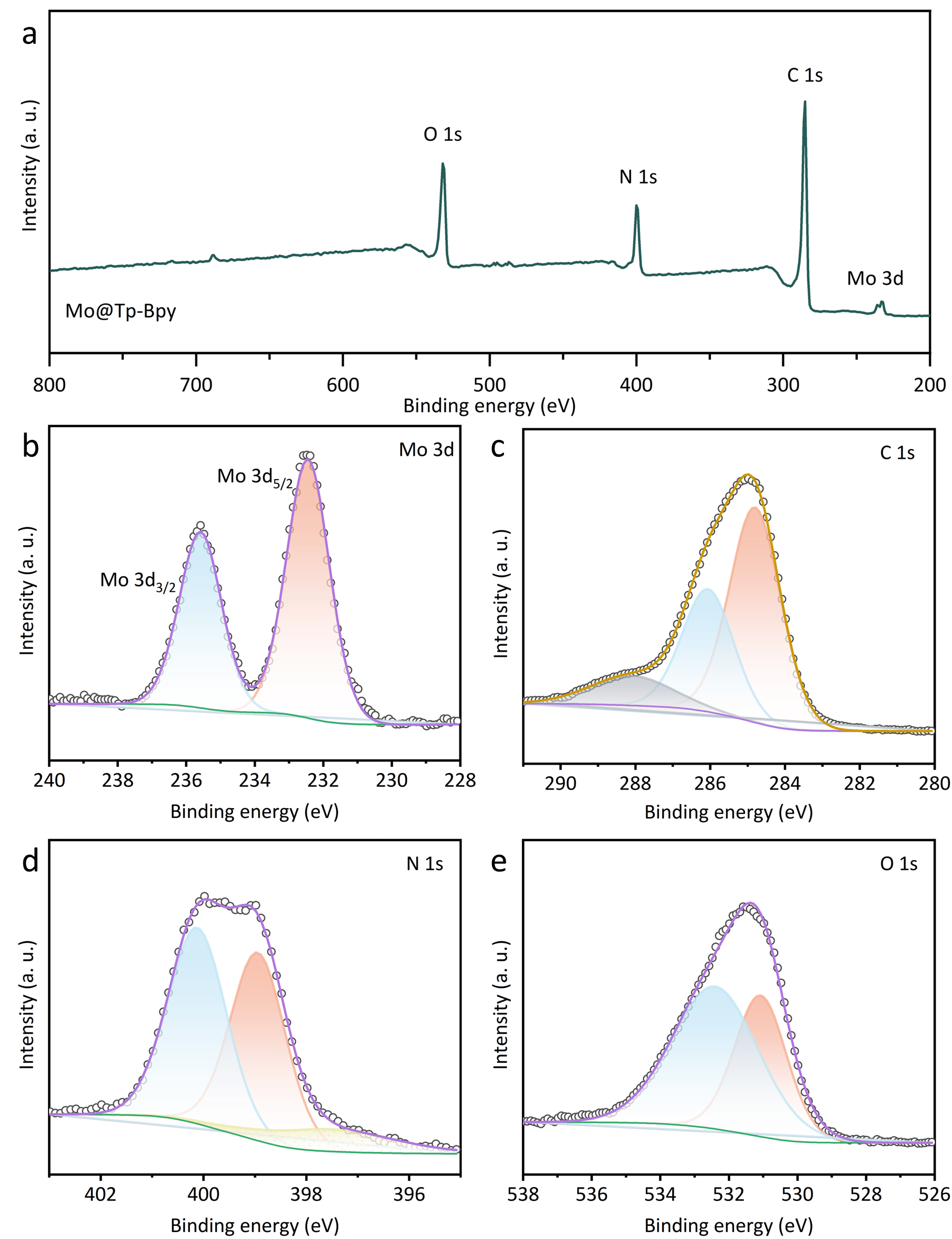


Figure S24. XPS spectra of (a) survey, (b) Mo 3d, (c) C 1s, (d) N 1s, and (e) O 1s for recycled Mo@Tp-Bpy.

**Note:** XPS analysis reveals that the Mo content changes from 0.73 at% before the reaction to 0.67 at% afterward, indicating negligible Mo leaching. Moreover, the Mo binding energy remains identical before and after the reaction, demonstrating that no change in oxidation state occurs.


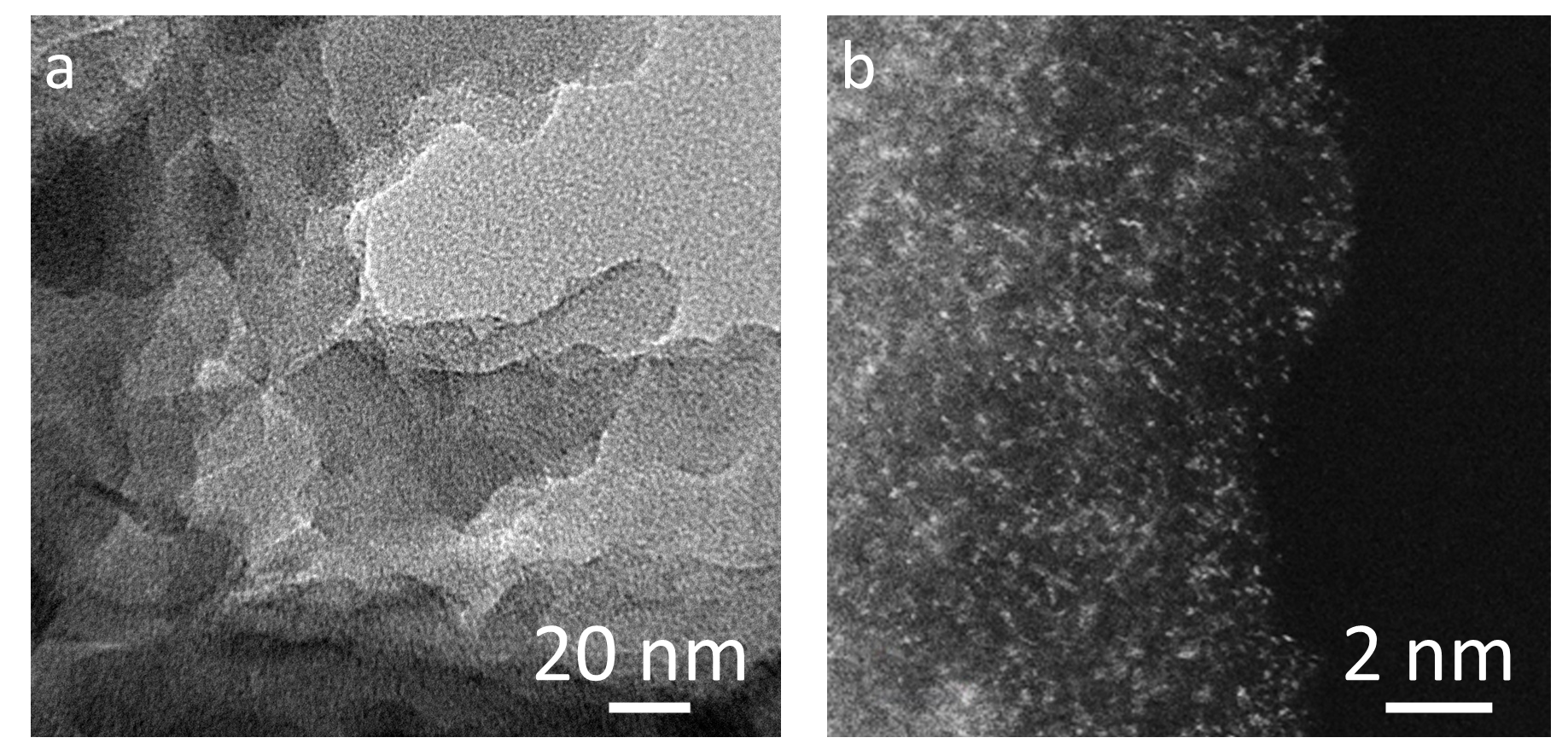


Figure S25. (a) TEM and (b) AC-HAADF-STEM images of Mo@Tp-Bpy after the durability test.


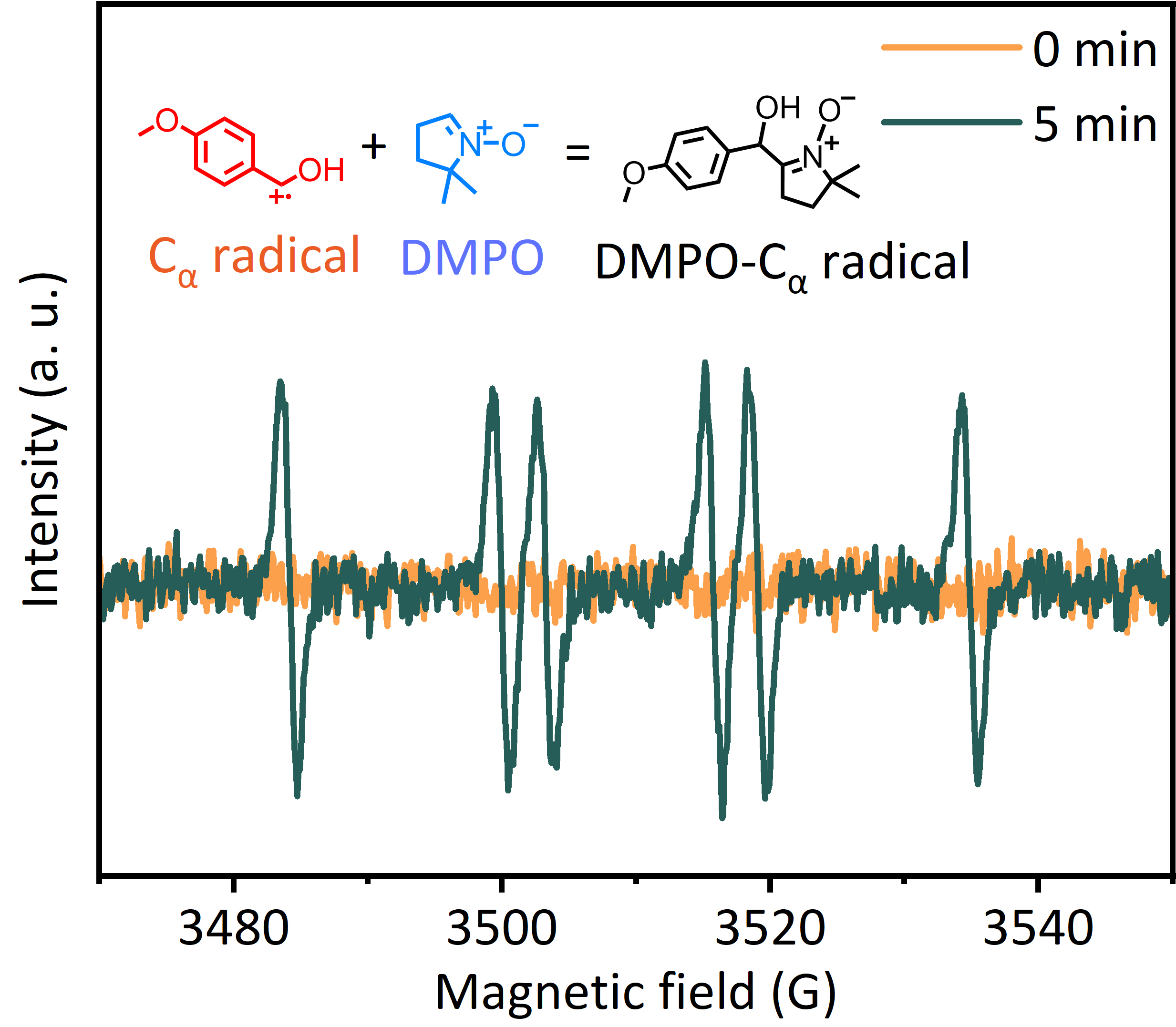


Figure S26. *In situ* DMPO-trapped ESR spectra for Mo@Tp-Bpy in 0.1mol/L 4-MBA solution.


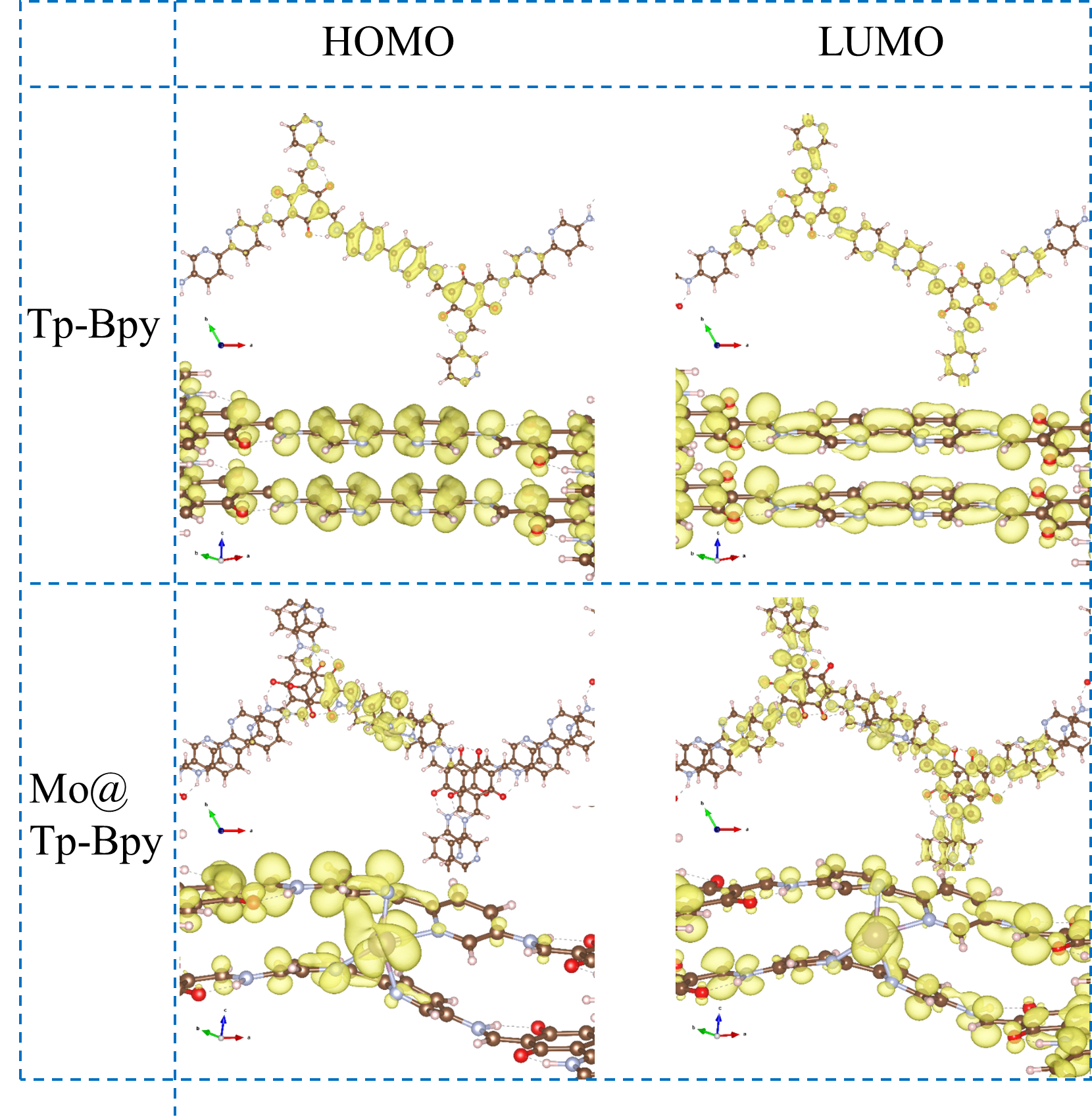


Figure S27. Partial charge density distributions for the HOMO and LUMO of Tp-Bpy and Mo@Tp-Bpy.


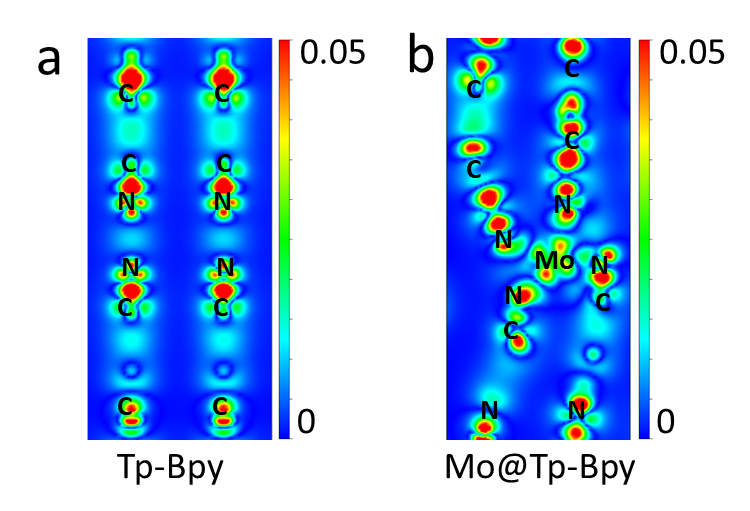


Figure S28. 2D charge density difference maps (unit in e/Bohr^2^) of (a) Tp-Bpy and (b) Mo@Tp-Bpy.


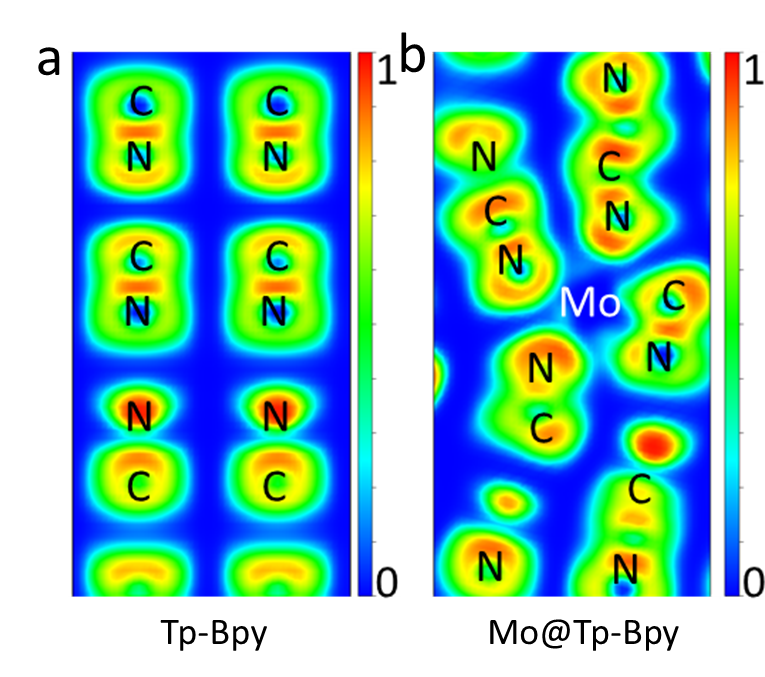


Figure S29. 2D mapping of ELF (ranging from 0 to 1, blue → red) of Tp-Bpy and Mo@Tp-Bpy.


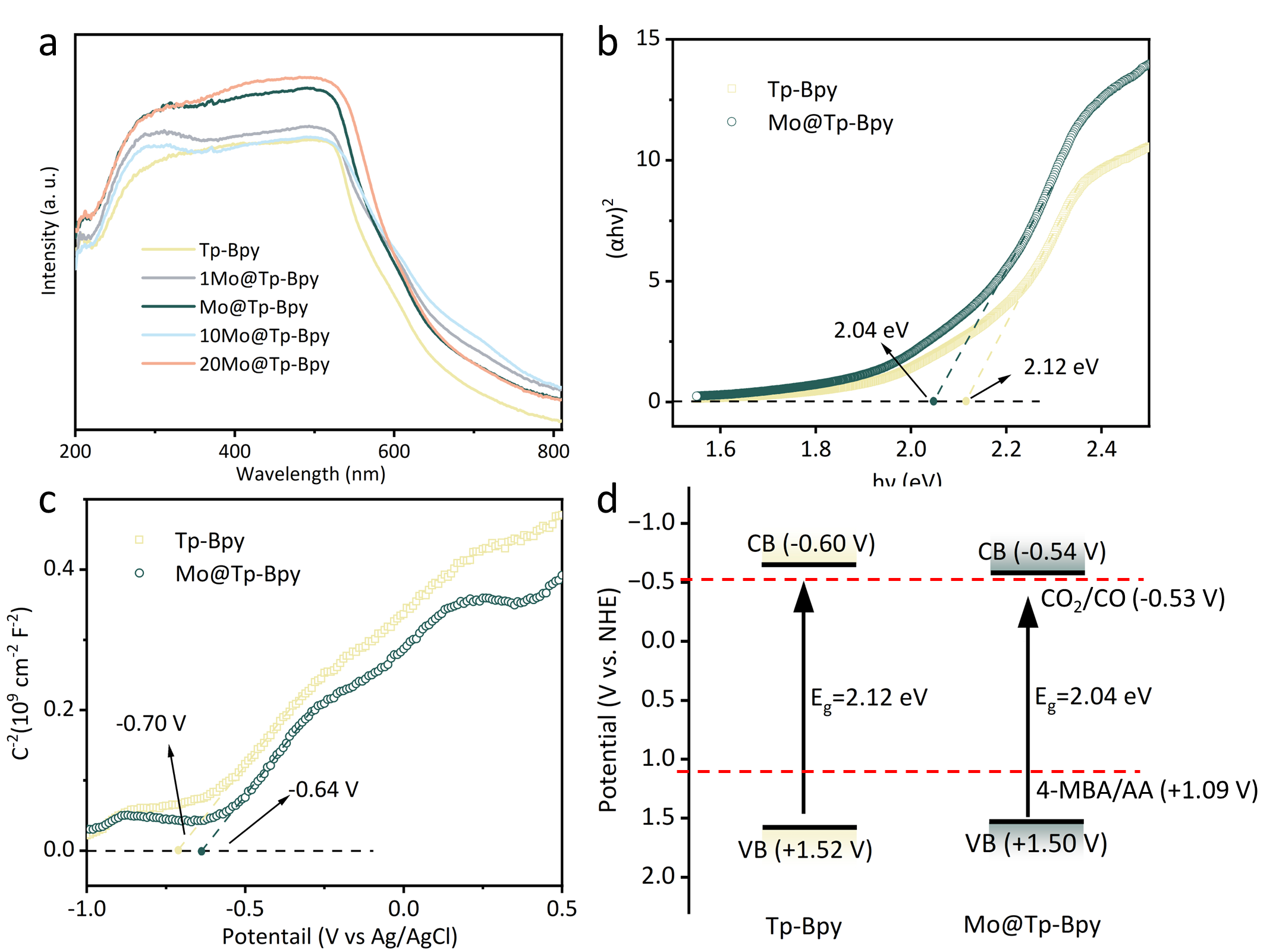


Figure S30. (a) UV-vis spectra, (b) Tauc plots, (c) M-S plots, and (d) band structures of different samples.

Note: The UV-Vis absorption spectra (**Figure S30a**) reveal a progressive red-shift of the absorption edge upon increasing Mo incorporation, suggesting effective modulation of the electronic structure. This behavior originates from the hybridization between Mo d-orbitals and the π-conjugated framework of Tp-Bpy, which reduces the energy barrier for optical transitions and extends the light-harvesting range. The corresponding Tauc plots (**Figure S30b**) yield optical bandgaps of 2.12 eV for pristine Tp-Bpy and 2.04 eV for Mo@Tp-Bpy. This bandgap narrowing is attributed to the introduction of mid-gap states derived from Mo d-orbitals, which create a "stepped" transition pathway between the HOMO and the LUMO. Such a configuration not only promotes the generation of photogenerated charge carriers but also balances carrier lifetime.

Mott-Schottky measurements (**Figure S30c**) exhibit positive slopes, confirming the n-type semiconductor nature of both materials. The flat-band potentials (V_fb_) are estimated at -0.7 V and -0.64 V (vs. Ag/AgCl, pH = 6.8), corresponding to -0.5 V and -0.44 V vs. NHE, respectively. Based on the typical expectation that the LUMO level of an n-type semiconductor lies 0.1-0.3 V more negative than V_fb_, the LUMO levels of Tp-Bpy and Mo@Tp-Bpy are calculated as -0.6 V and -0.54 V (vs. NHE), respectively.


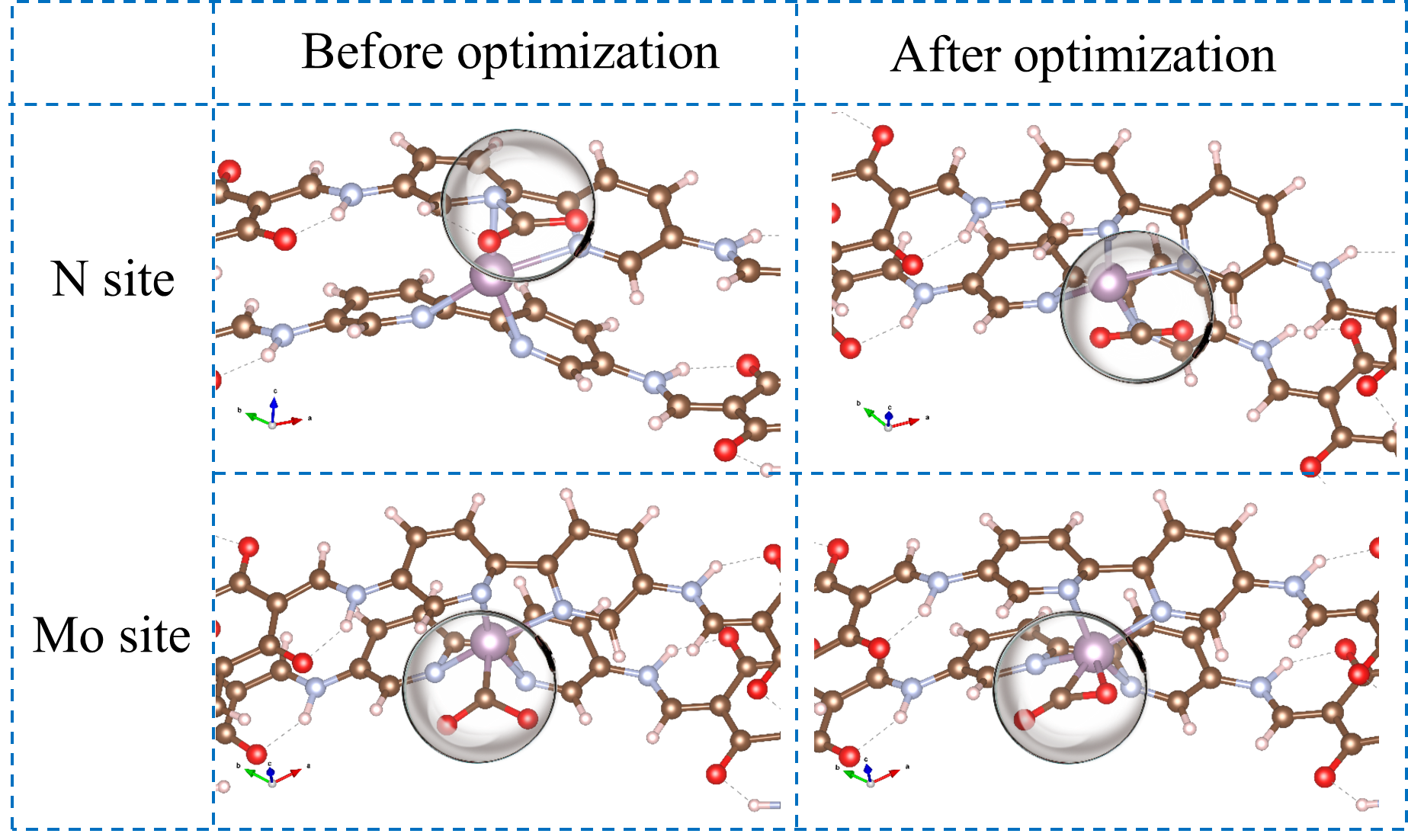


Figure S31. Comparison of CO_2_ adsorption configuration at different potential sites of Mo@Tp-Bpy surface.

Note: After structural optimization, CO_2_ desorbs from N sites, indicating their diminished direct adsorption role; instead, they serve as electron-transfer mediators. In contrast, at Mo single-atom sites, CO_2_ adopts a bidentate coordination involving both C and O atoms, accompanied by significant geometric bending and electronic rearrangement.


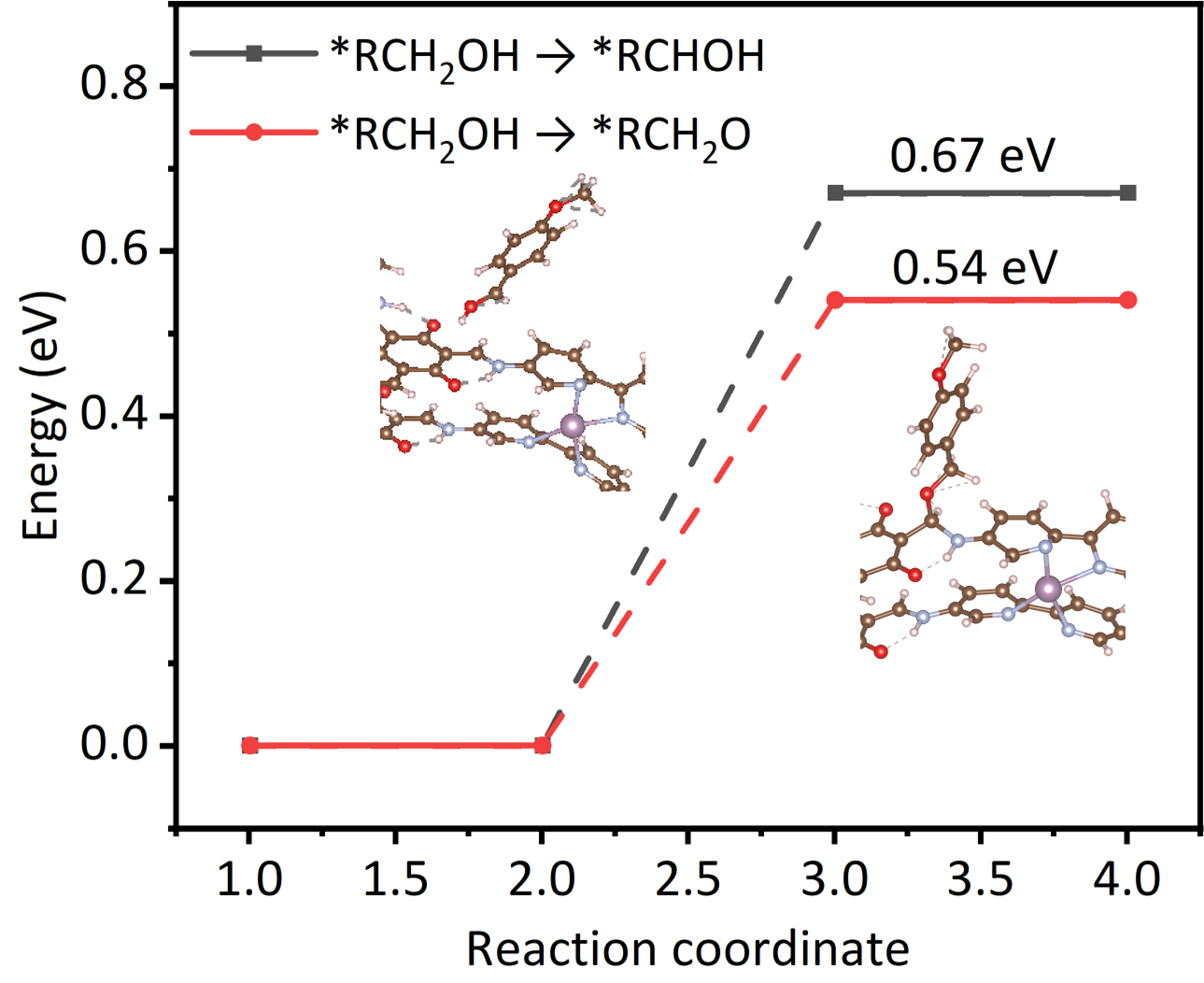


**Figure S32.** Comparison of calculated free energies for C–H bond and O–H bond cleavage of adsorbed-MBA over Mo@Tp-Bpy surface.

# Supplementary Tables

Table S1. Key porosity parameters of different samples.

| Samples | BET (m^2^ g^-1^) | *D*_micro_ (nm) | *V*_total_ (cm^3^ g^-1^) |
| --- | --- | --- | --- |
| Tp-Bpy | 780.3 | 1.6 | 0.31 |
| Mo@Tp-Bpy | 536.5 | 1.6 | 0.21 |

Note: Calculated from N_2_ adsorption-desorption isotherms by the BET method; the average diameters of mesopores and micropores are determined by the BJH and NLDFT methods, respectively.

Table S2. The content of Mo on Tp-Bpy in the XPS spectra collected before Ar+ sputtering (0 s) and at different times after sputtering (60 s and 120 s).

| Sputtering time (s) | | C (%) | N (%) | O (%) | Mo (%) |
| --- | --- | --- | --- | --- | --- |
| 0 | 42.71 | | 19.43 | 22.99 | 14.87 |
| 60 | 43.92 | | 20.88 | 18.59 | 16.61 |
| 120 | 46.61 | | 18.79 | 16.83 | 17.77 |

Note: The XPS depth profiling data reveal a gradual increase in Mo atomic concentration from 14.87% at the surface (0 s) to 17.77% after 120 s of Ar^+^ sputtering, while the C and N contents remain relatively stable. This trend indicates a genuine enrichment of Mo in the bulk rather than an artifact of preferential sputtering of the COF hydrocarbon backbone. The decrease in O content is primarily attributed to the removal of surface‑adsorbed oxygen species.

Table S3. EXAFS fitting parameters at the Mo K–edge for various samples.

| Sample | Path | N | R (Å) | σ^2^ (🞨10^-3^ Å^2^) | E_0_ (eV) | R-factor |
| --- | --- | --- | --- | --- | --- | --- |
| Mo foil | Mo-Mo | 8 | 2.72  (± 0.005) | 3.93 (± 0.70) | -1.83  (± 0.78) | 0.002 |
|  | Mo-Mo | 6 | 3.13  (± 0.006) | 3.41 (± 0.98) |  |  |
| Mo@Tp-Bpy | Mo-N | 4.10  (± 0.32) | 1.77  (± 0.01) | 3.56 (± 1.06) | 9.24  (± 1.27) | 0.010 |
| MoO_3_ | Mo-O | 1.95  (± 0.23) | 1.65  (± 0.01) | 5.21 (± 2.88) | 7.82  (± 3.47) | 0.012 |
|  | Mo-O | 3.74  (± 0.29) | 2.24  (± 0.02) |  |  |  |
|  | Mo-Mo | 1.97  (± 0.44) | 2.62  (± 0.04) | 7.34 (± 4.52) |  |  |
|  | Mo-Mo | 2.17  (± 0.37) | 3.93  (± 0.04) |  |  |  |

Note: *^a^CN*, coordination number; *^b^R*, distance between absorber and backscatter atoms; *^c^σ*^2^, Debye-Waller factor to account for both thermal and structural disorders; *^d^ΔE*_0_, inner potential correction; *R* factor indicates the goodness of the fit. S_0_^2^ was fixed to 0.75, according to the experimental EXAFS fit of Mo foil by fixing CN as the known crystallographic value. A reasonable range of EXAFS fitting parameters: 0.600 < *Ѕ*_0_^2^ < 1.000; *CN >* 0; *σ*^2^ > 0 Å^2^; |Δ*E*_0_| < 15 eV; *R* factor < 0.02.

Table S4. Summary of the photoluminescence decay time (*τ*) and their pre-exponential factor (*B*) of Tp-Bpy and Mo@Tp-Bpy suspensions.

| Sample | Conditions | Decay time (ns) | | | Pre-exponential factor (%) | | | τ_ave_(ps) |
| --- | --- | --- | --- | --- | --- | --- | --- | --- |
|  |  | *τ_1_ (ps)* | *τ_2_ (ps)* | *τ_3_ (ps)* | *B_1_* | *B_2_* | *B_3_* |  |
| Tp-Bpy | N_2_ | 0.31 | 3.27 | 18.65 | 10.71 | 86.5 | 2.79 | 5.61 |
|  | CO_2_ | 0.28 | 3.02 | 15.27 | 9.69 | 87.19 | 3.12 | 4.85 |
|  | 4-MBA | 0.36 | 3.33 | 20.14 | 8.73 | 86.96 | 4.31 | 7.15 |
| Mo@  Tp-Bpy | N_2_ | 0.28 | 3.06 | 19.44 | 19.35 | 77.33 | 3.33 | 6.47 |
|  | CO_2_ | 0.27 | 2.92 | 12.96 | 10.42 | 85.5 | 4.08 | 4.63 |
|  | 4-MBA | 0.43 | 3.05 | 16.56 | 24.16 | 66.92 | 8.91 | 8.48 |

Note: The formula of average lifetime is: *τ*_ave_ = (*B*_1_*τ*_1_^2^ + *B*_2_*τ*_2_^2^ + *B*_3_*τ*_3_^2^) / (*B*_1_*τ*_1_ + *B*_2_*τ*_2_ + *B*_3_*τ*_3_).

Table S5. The detailed quantitative data for the calibration curve in **Figure S21**.

| AA concentration (mmol/mL) | | Peak area  (a. u.) |
| --- | --- | --- |
| 0.00040098 | 26008 | |
| 0.00080196 | 52425 | |
| 0.00120295 | 76312 | |
| 0.00160393 | 103526 | |
| 0.00200492 | 128294 | |

Table S6. Comparison of Ti-MOF@TB-COF with the previously reported works on CO_2_ reduction coupled with selective of organics oxidation.

| Entry | | Catalyst | Reaction conditions | Products evolution rate  (μmol g^-1^ h^-1^) | Ref. |
| --- | --- | --- | --- | --- | --- |
| 1 | | Mo@Tp-Bpy | 300 W Xe light  1 mL 4-MBA +9 mL H_2_O | CO: 948.0  AA: 3741.7 | This work |
| 2 | | Cu_2_O/Cu | 300 W Xe light (AM1.5G)  Pure BA | CO: 34  HB: 62 | [9] |
| 3 | | CsPbBr_3_@PANI | 300 W Xe light  0.1 mL BA +20 mL CH_3_CN | CO: 26.1  BAD: 27.0 | [10] |
| 4 | | ZnIn_2_S_4_@CdS | 300 W Xe light  (𝜆 ≥ 400 nm)  BA +DMF | CO: 103.5  BAD: 176.6 | [11] |
| 5 | | FAPbBr_3_/Bi_2_WO_4_ | 150 W Xe light  AM 1.5  0.1 mmol BA | CO: 170.0  BAD: 250.0 | [12] |
| 6 | | In_2_O_3_/ZnIn_2_S_4_ | 300 W Xe light  (400 nm < λ < 800 nm)  10 mL BA +90 mL DMF | CO: 84.09  BAD: 160.0 | [13] |
| 7 | | Ni(OH)_2_/Zn_3_In_2_S_6_@ZIF-L-3 | 300 W Xe light  20 mL CH_3_CN + 0.8 mmol BA | CO: 69  BAD: 2312 | [14] |
| 8 | | Cs_3_Bi_2_Br_9_/W_18_O_49_ | 300 W Xe light  (𝜆 ≥ 420 nm)  5 mL toluene | CO: 177.4  BAD: 1702.3  BAD selectivity:81% | [15] |
| 9 | | W_18_O_49_/CsPbBr_3_ | 300 W Xe light  (𝜆 ≥ 420 nm)  5 mL toluene | CO: 143  BAD: 1546  BAD selectivity:80% | [16] |
| 10 | | CsPbBr_3_/TiO_2_ | 300 W Xe light  5 mL BF + 0.05 mmol BA | CO:78.1  BDA: 1770 | [17] |
| 11 | | CdSe/CdS | 300 W Xe light  10 mL CH_3_CN +0.1 mmol 4-MTP | CO:495  4-MPD: 1006  CO selectivity:55.2% | [18] |
| 12 | CN-S24 | 300 W Xe light  (𝜆 ≥ 420 nm)  5 mL toluene | CO: 425  BAD: 1162.5 | [19] |  |
| AA: anisaldehyde; BAD: benzaldehyde; BA: benzyl alcohol; 4-MTP: 4-methoxythiophenol; 4-MPD: bis(4-methoxyphenyl) disulfide; | | | | | |

# References

[1] G. Kresse, and J. Furthmüller, “VEfficient iterative schemes for ab initio total-energy calculations using a plane-wave basis set,” *Physical Review B*, 54 (1996): 11169-11186. https://doi.org/10.1103/PhysRevB.54.11169

[2] A. H. Larsen, J. J. Mortensen, J. Blomqvist, I. E. Castelli , R. Christensen, M. Dułak , J. Friis, M. N. Groves, B. Hammer, C. Hargus, E. D. Hermes, P. C. Jennings, P. B. Jensen, J. Kermode, J. R. Kitchin, E. L. Kolsbjerg, J. Kubal, K. Kaasbjerg, S. Lysgaard, J. B. Maronsson, T. Maxson, T. Olsen, L. Pastewka, A. Peterson, C. Rostgaard, J. Schiøtz, O. Schütt, M. Strange, K. S. Thygesen, T. Vegge, L. Vilhelmsen, M. Walter, Z. Zeng, and K. W. Jacobsen, “The atomic simulation environment—a Python library for working with atoms,” *Journal of Physics: Condensed Matter*, 29 (2017): 273002. https://doi.org/10.1088/1361-648X/aa680e

[3] G. Kresse, and D. Joubert, “From ultrasoft pseudopotentials to the projector augmented-wave method,” *Physical Review B*, 59 (1999): 1758-1775. https://doi.org/10.1103/PhysRevB.59.1758

[4] J. P. Perdew, K. Burke, and M. Ernzerhof, “Generalized Gradient Approximation Made Simple,” *Physical Review Letters*, 77 (1996): 3865-3868. https://doi.org/10.1103/PhysRevLett.77.3865

[5] S. Grimme, J. Antony, S. Ehrlich, and H. Krieg, “A consistent and accurate ab initio parametrization of density functional dispersion correction (DFT-D) for the 94 elements H-Pu,” *The Journal of Chemical Physics*, 132 (2010): 154104. https://doi.org/10.1063/1.3382344

[6] J. K. Nørskov, J. Rossmeisl, A. Logadottir, L. Lindqvist, J. R. Kitchin, T. Bligaard, and H. Jónsson, “Origin of the Overpotential for Oxygen Reduction at a Fuel-Cell Cathode,” *The Journal of Physical Chemistry B*, 108 (2004): 17886-17892. https://doi.org/10.1021/jp047349j

[7] G. Henkelman, B. P. Uberuaga, and H. Jónsson, “A climbing image nudged elastic band method for finding saddle points and minimum energy paths,” *The Journal of Chemical Physics*, 113 (2000): 9901-9904. https://doi.org/10.1063/1.1329672

[8] J. Pei, X. Chen, Y. Ning, and Q. Fu, “Visualizing dynamic evolution of surface electrochemical potential on solid-state electrolyte via spatially resolved photoelectron measurements,” *Nano Research*, 19 (2026): 94907863. https://doi.org/10.26599/NR.2025.94907863

[9] G. Ebri, E. Alhashmi, Y. Baghdadi, M. Daboczi, S. Eslava, and K. Hellgardt, “Simultaneous photocatalytic CO_2_ reduction and C-C coupling of benzyl alcohol under high pressure and supercritical conditions,” *Chemical Engineering Journal*, 505 (2025): 158356. https://doi.org/10.1016/j.cej.2024.158356

[10] F. Chen, Z. Li, Y. Jiang, Z. Li, R. Zeng, Z. Zhong, M.-D. Li, J. Z. Zhang, and B. Luo, “Photocatalytic CO_2_ Reduction Coupled with Oxidation of Benzyl Alcohol over CsPbBr3@PANI Nanocomposites,” *The Journal of Physical Chemistry Letters*, 14 (2023): 11008-11014. https://doi.org/10.1021/acs.jpclett.3c02766

[11] Y. Wang, J. Pu, J. An, X. Liang, W. Li, Y. Huang, J. Yang, T. Chen, and Y. Yao, “Tailoring Charge Separation in ZnIn_2_S_4_@CdS Hollow Nanocages for Simultaneous Alcohol Oxidation and CO_2_ Reduction under Visible Light,” *Inorganic Chemistry*, 63 (2024): 5269-5280. https://doi.org/10.1021/acs.inorgchem.4c00462

[12] H. Huang, J. Zhao, Y. Du, C. Zhou, M. Zhang, Z. Wang, Y. Weng, J. Long, J. Hofkens, J. A. Steele, and M. B. J. Roeffaers, “Direct Z-Scheme Heterojunction of Semicoherent FAPbBr_3_/Bi_2_WO_6_ Interface for Photoredox Reaction with Large Driving Force,” *ACS Nano*, 14 (2020): 16689-16697. https://doi.org/10.1021/acsnano.0c03146

[13] L. Li, J. Wang, K. Kuang, W. Ren, X. Zheng, S. Zhang, and S. Chen, “Construction of oxygen-deficient In2O3/ZnIn2S4 with hollow tubular heterostructure for photocatalytic CO2 reduction to syngas and benzyl alcohol oxidation,” *Separation and Purification Technology*, 330 (2024): 125527. https://doi.org/10.1016/j.seppur.2023.125527

[14] J. Chen, M. Mu, Z. Wang, M. Ma, F. A. Qaraah, X. Yin, and G. Bai, “Ni(OH)_2_-Decorated Zn_3_In_2_S_6_@ZIF-L Dual-S-Scheme Heterostructure for Cooperative Photocatalytic CO_2_ Reduction Coupling with Benzyl Alcohol Oxidation,” *ACS Sustainable Chemistry & Engineering*, 12 (2024): 18161-18173. https://doi.org/10.1021/acssuschemeng.4c07010

[15] M. Zhang, X. Zhang, Z. Zhang, L. Zhang, J. Liao, X. Zhang, C. Ge, and W. Zhou, “Lead-free perovskite Cs_3_Bi_2_Br_9_ quantum dots (QDs)/ultra-thin W_18_O_49_ nanobelts S-scheme heterojunction toward optimized photocatalytic CO_2_ reduction coupled with toluene oxidation,” *Chemical Engineering Journal*, 505 (2025): 159635. https://doi.org/10.1016/j.cej.2025.159635

[16] X. Jiang, Z. Chen, Y. Shu, A. M. Idris, S. Li, B. Peng, J. Wang, and Z. Li, “In-situ assembled S‑scheme heterojunction of CsPbBr3 nanocrystals and W18O49 ultrathin nanowires for enhanced bifunctional photocatalysis,” *Applied Catalysis B: Environment and Energy*, 348 (2024): 123840. https://doi.org/10.1016/j.apcatb.2024.123840

[17] X. Lv, D. Pan, S. Zheng, M. Zeeshan Shahid, G. Jiang, J. Wang, and Z. Li, “In-situ producing CsPbBr_3_ nanocrystals on (001)-faceted TiO_2_ nanosheets as S‑scheme heterostructure for bifunctional photocatalysis,” *Journal of Colloid and Interface Science*, 652 (2023): 673-679. https://doi.org/10.1016/j.jcis.2023.07.174

[18] Y. Zhang, L.-H. Gao, M.-Y. Qi, Z.-R. Tang, and Y.-J. Xu, “Cooperative photoredox coupling of CO_2_ reduction with thiols oxidation by hybrid CdSe/CdS semiconductor quantum dots,” *Applied Catalysis B: Environment and Energy*, 367 (2025): 125118. https://doi.org/10.1016/j.apcatb.2025.125118

[19] C. Qiu, S. Wang, J. Zuo, and B. Zhang, “Photocatalytic CO_2_ Reduction Coupled with Alcohol Oxidation over Porous Carbon Nitride,” *Catalysts*, 12 (2022): 672. https://doi.org/10.3390/catal12060672
